# CtIP-BRCA1 complex and MRE11 maintain replication forks in the presence of chain terminating nucleoside analogs

**DOI:** 10.1093/nar/gkz009

**Published:** 2019-01-18

**Authors:** Mohiuddin Mohiuddin, Md Maminur Rahman, Julian E Sale, Christopher E Pearson

**Affiliations:** 1 Program of Genetics and Genome Biology, The Hospital for Sick Children, Toronto, ON M5G 0A4, Canada; 2 Department of Radiation Genetics, Kyoto University Graduate School of Medicine, Yoshida Konoe, Sakyo-ku, Kyoto 606-8501, Japan; 3 Medical Research Council Laboratory of Molecular Biology, Hills Road, Cambridge CB2 0QH, UK; 4 The Department of Molecular Genetics, University of Toronto, Toronto, ON M5S 1A8, Canada

## Abstract

Chain-terminating nucleoside analogs (CTNAs), which cannot be extended by DNA polymerases, are widely used as antivirals or anti-cancer agents, and can induce cell death. Processing of blocked DNA ends, like camptothecin-induced trapped-topoisomerase I, can be mediated by TDP1, BRCA1, CtIP and MRE11. Here, we investigated whether the CtIP-BRCA1 complex and MRE11 also contribute to cellular tolerance to CTNAs, including 2’,3’-dideoxycytidine (ddC), cytarabine (ara-C) and zidovudine (Azidothymidine, AZT). We show that *BRCA1^−/−^, CtIP^S332A/−/−^* and nuclease-dead *MRE11^D20A/−^* mutants display increased sensitivity to CTNAs, accumulate more DNA damage (chromosomal breaks, γ-H2AX and neutral comets) when treated with CTNAs and exhibit significant delays in replication fork progression during exposure to CTNAs. Moreover, *BRCA1^−/−^, CtIP^S332A/−/−^* and nuclease-dead *MRE11^D20A/−^* mutants failed to resume DNA replication in response to CTNAs, whereas control and *CtIP^+/−/−^* cells experienced extensive recovery of DNA replication. In summary, we provide clear evidence that MRE11 and the collaborative action of BRCA1 and CtIP play a critical role in the nuclease-dependent removal of incorporated ddC from replicating genomic DNA. We propose that BRCA1-CTIP and MRE11 prepare nascent DNA ends, blocked from synthesis by CTNAs, for further repair.

## INTRODUCTION

Homologous recombination is initiated at double strand breaks (DSBs) by resection, a process in which DSB ends are converted into 3′-single-strand DNA overhangs. BRCA1 and CtIP play a critical role in facilitating DSB resection by the DNA2, EXO1 and MRE11 nucleases. Interaction of BRCA1 with CtIP is promoted by phosphorylation of Ser327 of CtIP by cyclin-dependent kinase 1 (CDK1) (1). This finding suggested the attractive idea that the BRCA1–CtIP interaction is involved in DSB resection. Replacement of this key serine in chicken DT40 cells (Ser332) (2) or in mice (3) resulted in a protein that failed to bind BRCA1, but which is fully capable of performing DSB resection and DSB repair by HR. Thus, the BRCA1–CtIP interaction is dispensable for DSB resection. However, the *CtIP^S327A^* mutation causes significant increases in cellular sensitivity to camptothecin (2), a Top1 poison, which stabilizes Top1-DNA-cleavage complex (Top1cc), a single-strand break (SSB) covalently associated with Top1 at the 3’ end of the break (4,5). Moreover, *BRCA1^−/−^* and *BRCA1^−/−^/CtIP^S332A/−/−^* DT40 cells show very similar sensitivity to camptothecin (2). These observations suggest that the BRCA1–CtIP complex facilitates removal of Top1 from Top1cc, a role played by Tyrosyl DNA phosphodiesterase 1 (TDP1), releasing Top1 together with covalently attached oligonucleotide. Since TDP1 can also eliminate incorporated chain terminating nucleoside analogs (6), an interesting question is whether the BRCA1–CtIP complex can also facilitate the removal of nucleoside analogs from the 3’ end of oligonucleotides.

Nucleoside analogs have been widely used for treating cancer and virus infection. Anti-viral nucleoside analogs, including Abacavir (ABC), Zidovudine (Azidothymidine, AZT), 2’3’ di-deoxycytidine (ddC) (7), are imported by cells, tri-phosphorylated, and incorporated by the viral DNA polymerases. These agents inhibit further extension by polymerases, leading premature termination of virus genome synthesis (8). Although anti-viral CTNAs are incorporated by viral DNA/RNA polymerases considerably more efficiently than by the replicative DNA polymerases of host cells (7,9), substantial numbers of anti-viral CTNAs might be mis-incorporated by the host polymerases, since the size of human genome is about five orders magnitudes larger than an average sized retrovirus genome. In fact, Abacavir is used for treating adult T cell leukemia, since malignant cells are hypersensitive to Abacavir due to very limited expression of TDP1 in the malignant cells (10). Thus, in addition to anti-viral therapy, CTNAs are used for anti-cancer therapy. Cytarabine-Ocfosphate-Hydrate (Ara-C, cytarabine), the first line chemotherapy agent for acute myeloid leukemia for the past 40 years, is also categorized as a CTNA (11,12). Exposure of cells to Abacavir induces formation of RAD51 recombinase foci (10), suggesting that premature termination of DNA replication is inducing recombinogenic intermediates.

BRCA1 and MRE11 play multiple roles in genome maintenance. The roles of BRCA1 include the promotion of both DSB resection, an initial step of DSB repair by HR, as well as a signal transduction in DNA damage checkpoint (13). The role of BRCA1 in DSB resection is centrally important for genome maintenance. *BRCA1^−/−^* mice, which are deficient in DSB resection, exhibit embryonic lethality, while the restoration of DSB resection and HR by additional inactivation of 53BP1, a NHEJ factor, normalizes development (14). It remains unclear whether or not BRCA1, CtIP and MRE11 contribute to quick recovery from the stalling of replication forks caused by mis-incorporated chain-terminating nucleoside analogs. To explore this previously uncharacterized role of BRCA1 in genome maintenance, we have exploited the phenotype of *53BP1^−/−^/BRCA1^−/−^* cells as a separation-of-function mutant that allows us to explore resection-independent functions of BRCA1 and CtIP.

We show here that *53BP1^−/−^/BRCA1^−/−^* and *CtIP^S332A/−/−^* cells derived from the chicken DT40 cell line were more sensitive to Ara-C, ABC, AZT and ddC in comparison with a *wild-type* control. These observations indicated that BRCA1 and CtIP contribute to cellular tolerance to CTNAs and that this contribution is independent of their contribution to HR. *BRCA1^−/−^* and *BRCA1^−/−^/CtIP^S332A/−/−^* DT40 cells showed very similar sensitivity to nucleoside analogs, supporting a role for BRCA1–CtIP complex formation in cellular tolerance to CTNAs. Similarly, *MRE11^D20A/-^* (nuclease defective) DT40 cells were more sensitive to Ara-C, ABC and ddC in comparison with *wild-type* control cells. Moreover, *BRCA1^−/−^, CtIP^S332A/−/−^* and nuclease-dead *MRE11^D20A/−^* mutants accumulate more DNA damage when treated with ddC, leading to cell death. Molecular combing analysis indicates that *MRE11^D20A/−^, BRCA1^−^*^*/−*^ and *CtIP^S332A/−/−^* DT40 mutants exhibit defects in the maintenance of replication fork progression following a 20 min pulse-exposure to ddC. Likewise, replication restart analysis indicates *BRCA1^−/−^, CtIP^S332A/−/−^* and nuclease-dead *MRE11^D20A/−^* mutants failed to resume DNA replication, whereas control and *CtIP^+/−/−^* cells experienced extensive recovery of DNA replication. We go on to show that the BRCA1–CtIP complex acts with the nuclease activity of MRE11 in resistance to CNTAs with *MRE11^D20A/−^* mutants exhibiting similar accumulation of DNA damage and cell death following exposure to Ara-C, ABC and ddC.

## MATERIALS AND METHODS

### Cell culture

DT40 and TK6 cells were cultured in RPMI 1640 medium (Nacalai Tesque Inc., Kyoto, Japan) as described previously (15,16). Supplementary Table S1 shows a list of gene-disrupted clones analyzed in this study, indicating the citations in which they have been characterized.

### Measurement of cellular sensitivity to DNA damaging agents

To measure sensitivity, cells were treated with olaparib (JS Research Chemicals Trading, Germany), camptothecin (Topogen, Inc, US) and several chain terminators such as ABC (Carbosynth, UK), Ara-C (Sigma, USA), AZT (Sigma, USA) and ddC (Sigma, USA). Cell sensitivity to these DNA-damaging agents and chain terminators was evaluated by counting colony formation in methylcellulose plates as described previously (17). In a liquid-culture cell survival assay, DT40 and TK6 cells were treated with DNA-damaging agents in 1 ml of medium using 24-well plates and incubated at 37°C for 72 h (DT40) or 96 h (TK6). We transferred 100 μl of medium containing cells to 96-well plates and measured the amount of ATP using cellTiter-Glo (Promega), according to the manufacturer's instructions. Relative cellular sensitivity to Ara-C, ABC, AZT and ddC was measured with methylcellulose colony formation. Briefly, to evaluate the relative cellular sensitivity of each mutant to *wild-type* cells, sensitivity curves were drawn by setting the survival of untreated cells as 100%. The concentration of 50% viability (inhibition concentration 50%; IC_50_) was determined from the sensitivity curves. The values of the mutant and *wild-type* cell lines were converted to a logarithmic scale (base 2). Each value was plotted on a bar graph.

### Measurement of RAD51 foci

Immunostaining analysis for DT40 cells has been described previously (18). Cells were applied to a glass slide using a cytospin and fixed using 4% paraformaldehyde for 10 min at room temperature and rinsed with PBS. Cells were permeabilized for 10 min in 0.1% NP40/PBS (Nonidet P-40) and rinsed with PBS. After blocking with 3% BSA/PBST, the cells were treated with specific primary anti-Rad51 monoclonal mouse antibodies (1:500, Millipore, Billerica, MA) for 60 min under humidified conditions at 37°C, followed by secondary Alexa 488-conjugated anti-mouse IgG antibodies (1:500, Molecular Probes, Eugene, OR) for 40 min and were thoroughly washed with PBS. The nuclei of at least 100 morphologically intact cells were examined for each group and the numbers of Rad51 foci were counted using fluorescence microscopy.

### Measurement of γH2AX foci

To evaluate the differential induction of DNA double- strand breaks by ddC in the DT40 strains, we determined the number of γH2AX foci in nuclear DNA. Chicken DT40 cells were suspended in culture medium at 1 × 10^6^ cells/ml and cultured in the presence or absence of 100 μM ddC for 9 h at 37°C. Cells were applied to a glass slide using a cytospin and fixed using 4% paraformaldehyde for 10 min at room temperature and rinsed with PBS. Cells were permeabilized for 10 min in 0.1% NP40/PBS (Nonidet P-40) and rinsed with PBS. After blocking with 3% BSA/PBST, the cells were treated with specific primary anti-γH2AX monoclonal mouse antibodies (1:500, Millipore, Billerica, MA, USA) for 60 min under humidified conditions at 37°C, followed by secondary Alexa 488-conjugated anti-mouse IgG antibodies (1:1000, Molecular Probes, Eugene, OR, USA) for 40 min and were thoroughly washed with PBS. The nuclei of at least 100 morphologically intact cells were examined for each group and the numbers of γH2AX foci were counted using fluorescence microscopy.

### Chromosomal aberration analysis

Chicken DT40 cells were suspended in culture medium at 1 × 10^6^ cells/ml and cultured in the presence or absence of 100 μM ddC for 9 h at 37°C. The cells were treated with 0.1 μg/ml colcemid (GIBCO-BRL, Grand Island, NY, USA) for the last 3 h before being harvested. Experimental conditions for chromosomal aberration analysis were as described previously (19). Briefly, harvested cells were treated with 1 ml of 75 mM KCl for 15 min at room temperature and fixed in 5 ml of a freshly prepared 3:1 mixture of methanol/acetic acid. The cell suspension was dropped onto an ice-cold wet glass slide and air-dried. The slides were stained with 5% Giemsa solution for 10 min and air-dried after being rinsed carefully with water. A total of 50 mitotic cells were scored for each group.

### Neutral comet assay for DSB detection

Chicken DT40 cells were cultured in the presence or absence of 4 mM ddC for 2 h at 37°C. After 2 h, a total of 700 cells were resuspended in 70 μl 0.5% low melting point agarose (Trevigen, 4250-050-02) at a ratio of 1:10 (v/v) and immediately spread on a comet slide (Trevigen, 4250-200-03). Slides were placed flat at 4°C in the dark for 30 minutes. Cells were lysed in a cold lysis solution (Trevigen, 4250-050-01) at 4°C for 1 h. DNA migration was performed in TBE buffer at 1 V cm^−1^ for 30 min. Slides were washed in milliQ water for 5 min and then fixed with 70% ethanol for 30 min and dried at room temperature. Comets were labeled with SYBR^®^ Green Nucleic Acid Gel Stain (ThermoFisher) for 30 min at room temperature in the dark. Images were acquired with a confocal fluorescence microscope and analyzed using ImageJ (Open Comet) software. At least 150 comets were scored per sample in each experiment.

### Dynamic molecular combing and immunofluorescent detection

Asynchronously growing DT40 cells were sequentially labeled for 20 min with 25 μM IdU and for 20 min with 25 μM CIdU. Dideoxycytidine (ddC) treated cells were exposed to 2 mM ddC just before the CldU treatment. At the end of the labeling period (40 min), cells were placed in ice cold 1 × PBS (1 volume of cells for 2 volumes of 1 × PBS) and centrifuged at 250 g for 5 min at 4°C, washed in ice-cold PBS, and re-suspended in PBS to a final concentration of 1 × 10^6^ cells/ml. 3 μl of the cell suspension was spotted onto clean glass Superfrost slides and lysed with 7 μl of 0.5% SDS in 200 mM Tris–HCl (pH 5.5) and 50 mM EDTA (5 min, at room temperature). Slides were tilted at 15° to horizontal, allowing the DNA to run slowly down the slide. Slides were then air dried and fixed in 3:1 methanol/acetic acid, and stored at 4°C before immunolabelling. IdU, CldU revelations and analysis were performed as described (20–22), with minor modifications: the DNA was denatured for 30 min in 2.5 N HCl, and CldU was detected using rat anti BrdU (ABD Serotec, Raleigh, NC) at 1/750. A stretching factor of 2.6 for conversion from μm to kb was applied, as previously described for the method in (23). Slides were mounted in 10% 1× PBS and 90% glycerol, kept at −20°C and imaged using a Nikon C1-si confocal microscope.

### DNA replication restart assays

Replicative cells were marked by pulse labeling for 30 min with 50 μM of CldU and then arrested for 6 h by treating with 2 mM ddC or 2 mM HU. After drug removal, cells were incubated with 50 μM of IdU in fresh complete medium for 60 min so that replication restart after stalling could be visualized. All the following steps were carried out at room temperature. Cells were fixed in 4% paraformaldehyde for 10 min and then permeabilized in 0.5% Triton X-100 (for TK6 cells) or 0.1% NP-40 (for DT40 cells) for 20 min. Cells were incubated with 2 M HCl for 45 min to denature the DNA and then blocked for 1 h with 5% FCS in PBS. Cells were immunostained for 1 h with the first primary antibody of rat monoclonal anti-BrdU [BU1/75, Abcam], washed with 0.05% PBST20 and then immunolabeled for 40 min with goat anti-rat Alexa 488-conjugated secondary antibody (Molecular Probes) to label CldU. Then, cells were immunostained for 1 h with the second primary antibody of mouse monoclonal anti-BrdU (Becton Dickinson). Cells were subsequently labeled for 40 min with goat anti-mouse Alexa 594 conjugated secondary antibody (Molecular Probes) to label IdU. Replication restart is represented by the overlap of CldU and IdU. Nuclei were counterstained with DAPI as in immunofluorescence microscopy assays.

## RESULTS

### The BRCA1–CtIP complex contributes to cellular tolerance to various CTNAs, independently of its role in HR

**Figure 1.**
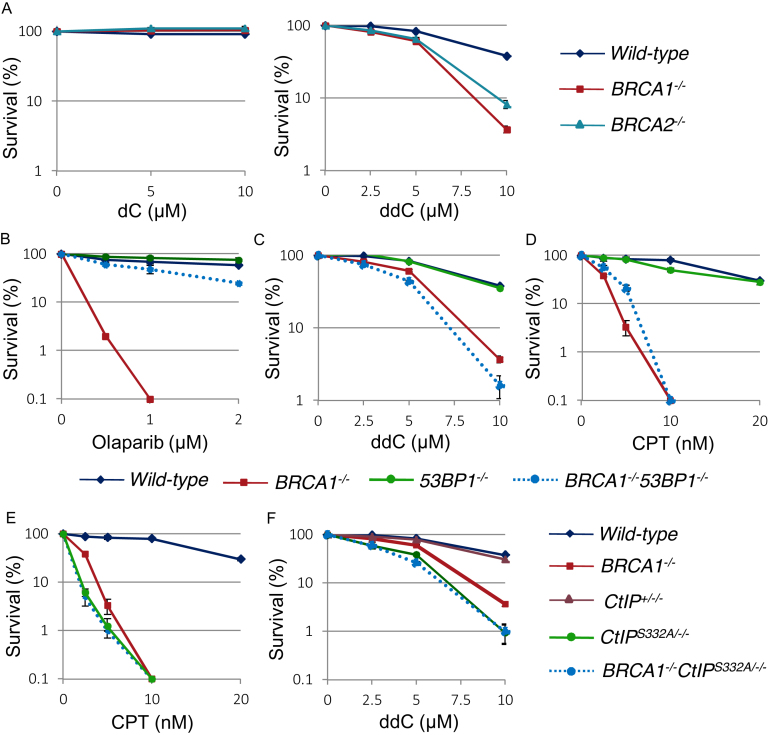
BRCA1 contributes to cellular tolerance to the CTNAs such as ddC, by the interaction with phosphorylated CtIP which is independent of its function in HR. (**A**) Cellular sensitivity of *BRCA1^−/−^* and *BRCA2^−^*^*/−*^ mutants to dC (left panel) and ddC (right panel) was analyzed. Survival rate was calculated as the percentage of surviving cells treated with DNA-damaging agents relative to the untreated surviving cells. The concentration or dose is displayed on the x-axis on a linear scale, while the survival rate is displayed on the y-axis on a logarithmic scale. Error bars show the standard error of the mean in at least three independent experiments. (B–D) Cellular sensitivity of *BRCA1^−/−^, 53BP1^−^*^*/−*^ and *BRCA1^−/−^/53BP1^−^*^*/−*^ mutants to olaparib (**B**), ddC (**C**) and CPT (**D**) was analyzed. Survival rate was calculated as Figure 1A. (E, F) Cellular sensitivity of *BRCA1^−/−^, CtIP*^*S*332*A/−/−*^ and BRCA1^−/−^/CTIP^*S*332*A/−/−*^ mutants to CPT (**E**) and ddC (**F**) was analyzed. Survival rate was calculated as Figure 1A.

BRCA1 and BRCA2 play key roles in HR (13). Based upon the activity of the BRCA1–CtIP complex in removing 3’ adducts at DSBs induced by camptothecin (2), we tested the possibility that BRCA1 may act in the removal of chain terminating nucleoside analogs. Taking advantage of viable *BRCA1^−/−^* and *BRCA2^−/−^* null mutant DT40 cells (24), we measured the sensitivity of these mutants to ddC. Supplementary Table S1 lists the mutant cells analyzed in this study. *BRCA2^−/−^* cells were sensitive to ddC (Figure 1A), an observation that agrees with the important role of HR in maintaining DNA replication fork progression (25,26). Although *BRCA1^−/−^* DT40 cells exhibit less prominent defects in HR than did *BRCA2^−/−^* cells (24), *BRCA1^−/−^* cells were considerably more sensitive to ddC in comparison with *BRCA2^−/−^* cells (Figure 1A). This observation is reminiscent of the activity of BRCA1–CtIP complex in removing 3’ adducts at DSBs induced by camptothecin, independent of the role of BRCA1 in HR (2). These observations suggest that BRCA1 may contribute to cellular tolerance to ddC independently of its function in HR.

To further address this HR-independent function of BRCA1, we analyzed *53BP1^−/−^/BRCA1^−/−^* DT40 cells (Supplementary Figure S1A–C). As expected, the loss of 53BP1 restored HR capacity in *BRCA1^−/−^* cells, as monitored by cellular resistance to olaparib, the poly[ADP ribose]polymerase (PARP) poison (27,28) (Figure 1B) and Rad51 focus formation following γ-irradiation (Supplementary Figure S1B and C). Remarkably, *53BP1^−/−^/BRCA1^−/−^* DT40 cells were still considerably sensitive to ddC as well as to camptothecin (Figure 1C and D), indicating the role of cellular tolerance to ddC was independent of the role of BRCA1 in HR. We next analyzed the sensitivity of *CtIP^S332A/−/−^, BRCA1^−/−^* and *BRCA1^−/−^/CtIP^S332A/−/−^* DT40 cells to camptothecin and ddC. Takeda's group previously showed the sensitivity of *CtIP^S332A^* to camptothecin as well as an epistatic relationship between the *CtIP^S332A/−/−^* and *BRCA1^−/−^* mutations in cellular sensitivity to camptothecin (2) as shown in Figure 1E. Phosphorylation of Ser332 residue of CtIP is critical for the interaction of CtIP with BRCA1 (2,3). *CtIP^S332A/−/−^* cells were more sensitive to ddC in comparison with the *CtIP^+/−/−^* control (Figure 1F). Moreover, *BRCA1^−/−^* and *BRCA1^−/−^/CtIP^S332A/−/−^* cells showed a similar sensitivity to ddC (Figure 1F). In summary, BRCA1 is involved in cellular tolerance to ddC independently of its role in HR, and its function depends on the physical interaction with CtIP.

**Figure 2.**
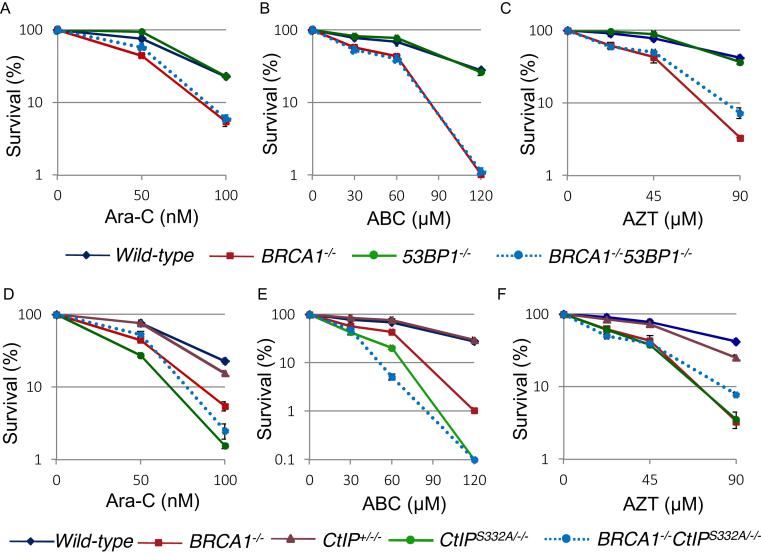
Important role of BRCA1 and CtIP for cellular tolerance to nucleoside analogs in DT40 cells. (**A–F**) Clonogenic cell survival to the indicated agents was analyzed as described in Figure 1A.

We next analyzed whether other CTNAs, ABC, Ara-C and AZT, had the same effect on *53BP1^−/−^/BRCA1^−/−^* and *CtIP^S332A/−/−^* DT40 cells, as did ddC. Both *53BP1^−/−^/BRCA1^−/−^* and *CtIP^S332A/−/−^* DT40 cells were considerably more sensitive to ABC, Ara-C and AZT in comparison with *wild-type* and *CtIP^+/−/−^* DT40 cells (Figure 2A to F). Moreover, *BRCA1^−/−^* and *BRCA1^−/−^/CtIP^S332A/−/−^* cells showed similar sensitivity to ABC, Ara-C and AZT. We then calculated IC_50_ (inhibitory concentration 50%) of these drugs, at which concentration the colony survival was decreased by half relative to untreated cells. Supplementary Figure S2 shows the ratio of IC_50_ of each isogenic mutant chicken DT40 cell lines relative to IC_50_ of *wild-type* cells on a logarithmic scale. We conclude that BRCA1 may be required for efficient recovery of DNA replication upon premature termination of DNA synthesis by CTNAs.

### Involvement of MRE11 in cellular tolerance to CTNAs

**Figure 3.**
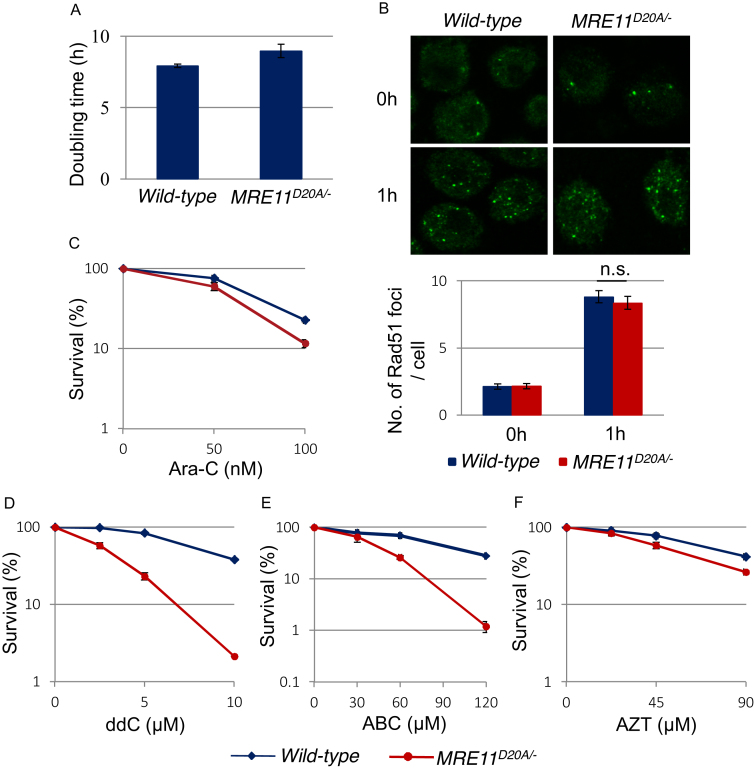
MRE11 is involved in cellular tolerance to CTNAs. (**A**) Cellular proliferation of *MRE11^D20A/−^* mutant cells. They proliferate almost the same as *wild-type* cells. (**B**) Accumulation of RAD51 at DNA damage sites is indistinguishable between *wild-type* and *MRE11^D20A/−^* mutant cells. Representative fluorescence microscopic images (upper panel) and quantification (lower panel) of Rad51 foci in the indicated cell lines before and 1 h after irradiation of 2 Gy IR. Statistical analysis was done by Student's t-test (n.s.: not significant). (**C–F**) Clonogenic cell survival to the indicated agents was analyzed as described in Figure 1A.

MRE11 shows 3’ to 5’ exonuclease activity in the presence of NBS1 (29), which agrees with the role of MRE11 in the removal of nucleotides from primer sequences when ddC blocks extension by replicative DNA polymerases. Based upon the 3’ to 5’ exonuclease activity of MRE11, we next explored the involvement of MRE11 in cellular tolerance to CTNAs. We used hypomorphic *MRE11*^*D20A/−*^ DT40 cells (Supplementary Figure S3), since the complete loss of MRE11 is lethal to cells (30). The Asp20 residue of vertebrate MRE11 corresponds to the Asp16 residue of MRE11 in *Saccharomyces cerevisiae* and localizes in the N-terminal phosphodiesterase motif. In yeast, the *MRE11^D16A^* mutation strongly reduces *in vitro* nuclease activities of MRE11 and causes the accumulation of unresected meiotic DSBs in *S. cerevisiae* (31). *MRE11*^*D20A/−*^ DT40 cells were able to proliferate, proficient in DSB resection as evidenced by normal RAD51 focus formation at 1h after ionizing-radiation (Figure 3A and B). Thus, the *MRE11^D20A^* point mutation has no impact on DSB resection or DSB repair by HR. However, *MRE11*^*D20A/−*^ DT40 cells were sensitive to Ara-C, ABC, AZT and ddC (Figure 3C-F and Supplementary Figure S2). These observations suggest that MRE11 contributes to cellular tolerance to CTNAs independent of its role in DSB resection.

We next explored the involvement of NBS1 in cellular tolerance to CTNAs. To this, we used a conditionally disrupted *NBS1^−/−/−^* gene in DT40 cells (32), since the complete loss of NBS1 is lethal to cells. *NBS1^−/−/−^* DT40 cells showed sensitivity to ddC and ABC (Supplementary Figure S4) indicating that NBS1 is also required for removing CTNAs from the replicative DNA.

### Increased DNA damage induced by ddC in *CtIP*^*S332A/−/−*^, *BRCA1^−/−^* and nuclease dead *MRE11* mutant cells

An inability to remove CTNAs might be expected to cause an increase in DNA damage. Phosphorylated histone H2AX (γH2AX) foci are hallmarks of double-strand breaks and replication fork stalling (33). To evaluate the induction of DNA damage by ddC, we measured the number of sub-nuclear γH2AX foci in the chicken DT40 cells. We defined γH2AX-foci positive cells as cells displaying more than seven foci per cell (Supplementary Figure S5), since the number of spontaneously arising γH2AX-foci did not exceed seven foci per cell in almost 90 percent of cells. The immunofluorescence analysis of γH2AX foci suggested that ddC significantly induced DNA damage in *wild-type* DT40 cells (Supplementary Figure S5). We conclude that mis-incorporation of ddC by replicative polymerases causes significant replication stress upon mis-incorporation that leads to cell death (Figure 1F). Consistently, the number of γH2AX foci per cell is also significantly increased in wild-type DT40 cells (Supplementary Figure S5). Remarkably, the phosphorylation deficient *CtIP* mutant, *CtIP^S332A/−/−^*, as well as *BRCA1^−/−^* and nuclease dead *MRE11* mutant cells displayed significantly higher number of γH2AX foci per cell and higher percentage of ddC- induced γH2AX-foci-positive cells immediately after ddC treatment in comparison with *wild-type* and *CtIP^+/−/−^* cells (Supplementary Figure S5), indicating that ddC induces a DNA damage response by interfering with DNA replication during its incorporation by replicative DNA polymerases.

**Figure 4.**
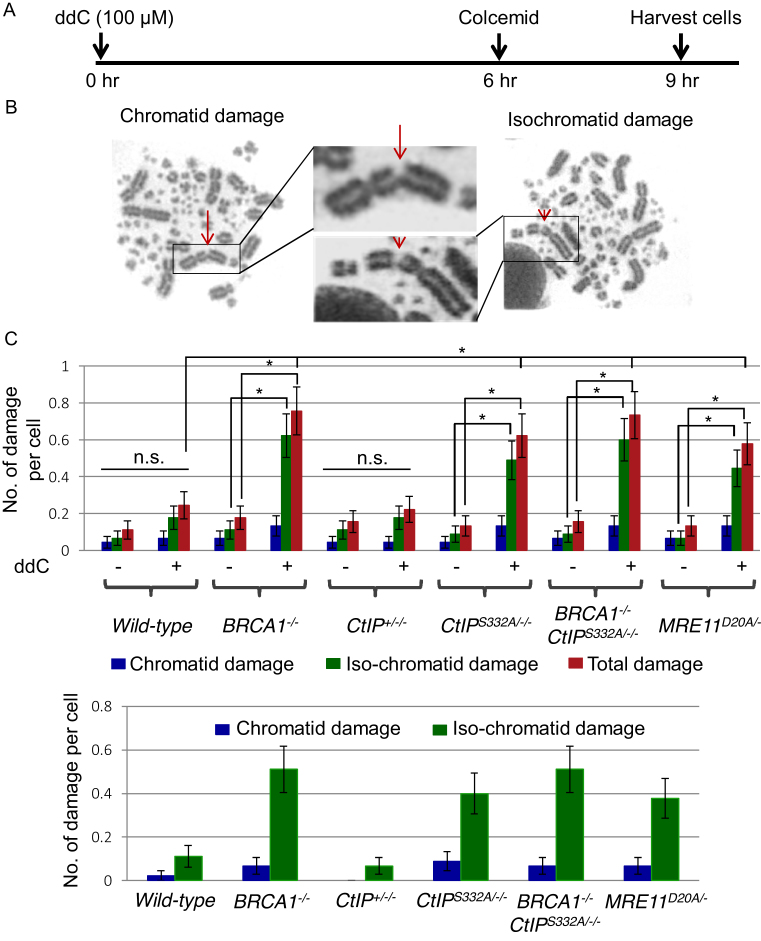
*BRCA1^−/−^, CtIP^S332A/−/−^* and nuclease-dead *MRE11^D20A/−^* mutants exhibit increased number of ddC-induced chromosomal aberrations. (**A**) Schematic representation of the experimental protocol to measure chromosomal aberrations. (**B**) Representative images of chromatid damage (arrow) and isochromatid damage (arrowhead) after ddC treatment. (**C**) Number of damage per mitotic cells in the indicated genotypes (Upper panel). Error bars represent standard deviation. At least 50 mitotic cells were counted for each cell line. The *P*-value for Student's *t*-test was *P< 0.01. The subtracted numbers of damages before the exposure from damages after the exposure (Lower panel). Error bars represent standard deviation.

To monitor the induction of DSBs, we measured the number of chromosomal aberrations in mitotic chromosome spreads at nine hours post-ddC treatment (Figure 4A). We counted the number of chromosome aberrations distinguishing chromatid breaks (one of the two sister chromatids is broken) and isochromatid breaks (two sister chromatids are broken at the same site) (Figure 4B). Data show that ddC induced significantly increased numbers of isochromatid type chromosomal breaks in *BRCA1^−/−^, CtIP^S332A/−/−^* and *MRE11^D20A/−^* cells, but not in *CtIP^+^*^*/−*^*^/−^* cells, in comparison with *wild-type* cells (Figure 4C), indicating that ddC affects chromosome integrity by inducing DNA damage through interfering with DNA replication during its incorporation by replicative DNA polymerases. The predominance of isochromatid breaks is consistent with damage arising during S-phase (34,35). These findings are consistent with previous reports of chromosomal aberrations induced by Abacavir, Ara-C and AZT (10,36,37).

**Figure 5.**
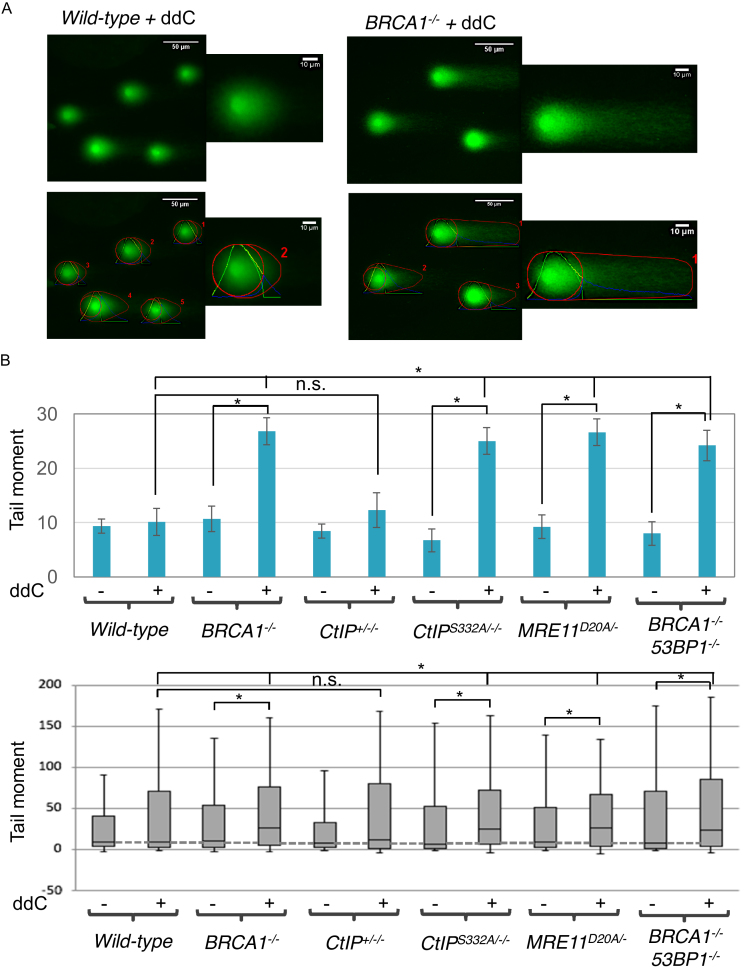
*BRCA1^−/−^, BRCA1^−/−^53BP1^−/−^, CtIP^S332A/−/−^* and nuclease-dead *MRE11^D20A/−^* mutants exhibit increased number of ddC-induced DNA double strand breaks. (**A**) Representative comet images (upper panel) and OpenComet output images (lower panel) of BRCA1-proficient and -deficient cells following treatment with 4 mM ddC for 2 h. (**B**) Neutral Comet assay monitoring DSB formation in *BRCA1^−/−^, BRCA1^−/−^53BP1^−/−^, CtIP^S332A/−/−^* and nuclease-dead *MRE11^D20A/−^* DT40 cells following ddC treatment for 2 h. The bar graph (upper panel) represents median and SD of comet tails. Out of three repeats; *n* ≥ 150 comets scored for each data set. The box and whisker plot (lower panel) represents median value of comet tails. Whiskers the 10th and 90th percentiles. *P< 0.01 (Student's *t*-test).

To verify an accumulation of DSBs following ddC treatment, we next measured DSBs in cells with or without ddC treatment by a comet assay under neutral conditions, in which DNA DSBs but not SSBs can be detected (38). Data show that ddC induced significantly increased number of DSBs in *BRCA1^−/−^, CtIP^S332A/−/−^* and *MRE11^D20A/−^* cells, but not in *CtIP^+^*^*/−*^*^/−^* cells, in comparison with *wild-type* cells (Figure 5 and Supplementary Figure S6), which is consistent with the increased DSBs detected by γ-H2AX foci levels or chromosomal breaks. Remarkably, *53BP1^−/−^/BRCA1^−/−^* DT40 cells show almost the same number of DSBs in comparison with BRCA*1^−/−^* cells (Figure 5 and Supplementary Figure S6), indicating its role in response to ddC was independent of the role of BRCA1 in HR.

All these characteristics, including the increased γ-H2AX foci levels (Supplementary Figure S5), increased number of isochromatid breaks (Figure 4) and elongated comet tail moments (Figure 5 and Supplementary Figure S6) indicate that phosphorylation deficient *CtIP* mutant as well as *BRCA1^−/−^* and nuclease dead *MRE11* mutant cells accumulate more DNA damage when treated with ddC, leading to increased cell death. These results suggest that, in addition to repairing CPT-induced damage (2), BRCA1–CtIP complex and MRE11 nuclease repair 3’ -blocking lesions induced by therapeutic ddC.

### BRCA1–CtIP and MRE11 are required to maintain replication fork progression in the face of premature termination by ddCTP

To analyze the impact of ddC on the progression of individual replication forks, we measured the kinetics of DNA replication using molecular combing (Supplementary Figure S7A). The replication rate of *BRCA1^−/−^, CtIP*^*S332A/−/−*^ and *MRE11*^*D20A/−*^ DT40 cells in unperturbed conditions was not significantly different in comparison with that of *wild-type* cells (Supplementary Figure S7B). Thus, the loss of BRCA1, CtIP and MRE11 does not attenuate global progression of DNA replication forks on undamaged DNA templates. We next examined replication fork progression following ddC treatment in the DT40 mutants.

**Figure 6.**
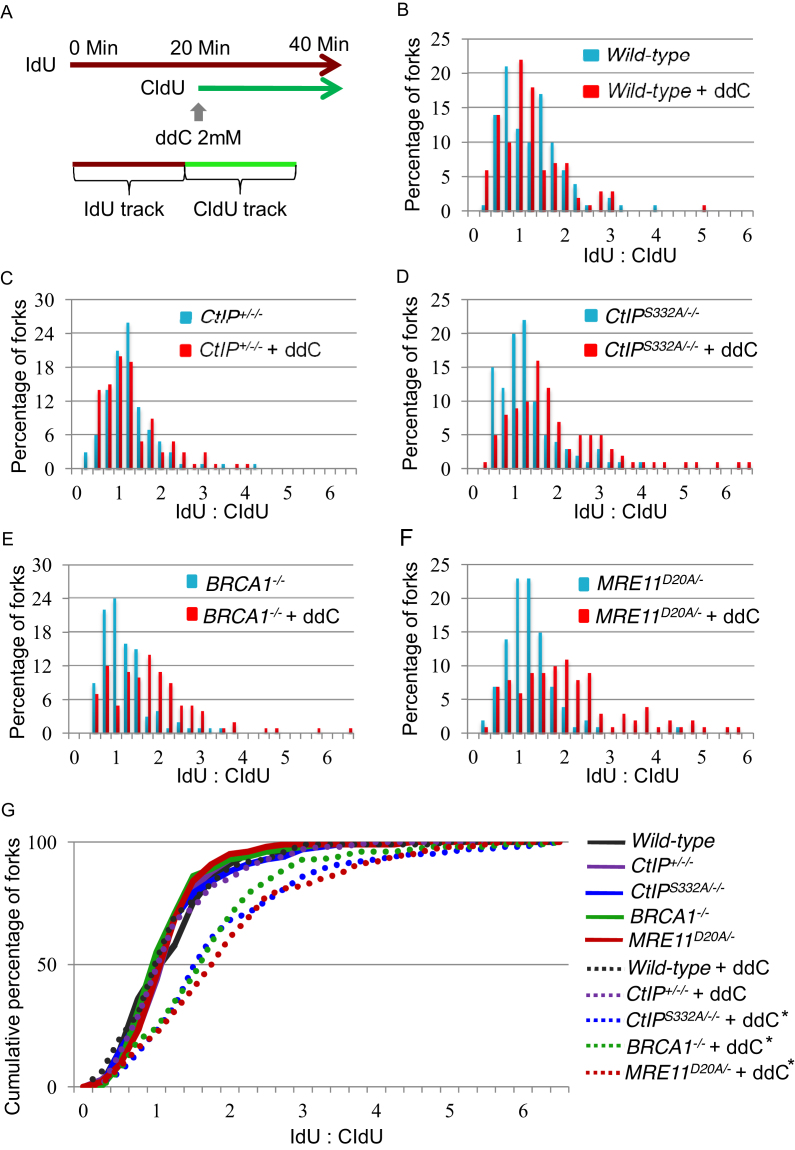
Phosphorylation deficient *CtIP* mutant but not *CtIP^+/−/−^* cells as well as *BRCA1^−/−^* and nuclease dead *MRE11* mutants exhibit defective progression of replication forks on ddc treatment. (**A**) Schematic of treatment with ddC and pulse labeling with IdU and CldU are shown. (B–F) Replication stalling in response to 2 mM ddC (red bars) or sham treatment (blue bars) in WT (**B**), *CtIP^+^*^*/−*^*^/−^* (**C**), *CtIP^S332A/−/−^* (**D**), *BRCA1^−/−^* (**E**) and *MRE11*^*D20A/−*^ (**F**) chicken DT40 cell lines. Each data set is derived from measurement of at least 100 forks. (**G**) The data for cells carrying the indicated genotypes was plotted as a cumulative percentage (y-axis) of forks at each ratio (x-axis). The P-values of the Kolmogorov–Smirnov test for ratio distribution of each mutant for UV compared to sham treatment are *P < 0.002 and **P < 0.001.

To analyze replication fork progression by DNA molecular combing, we labeled nascent strands with IdU for 20 min, exposed the cells to 2 mM ddC and then continued labeling the nascent strands with CldU for another 20 min (Figure 6A). After the DNA combing, we visualized the tracts of CldU and IdU, and calculated the ratio between them to compare the total DNA synthesized in the absence and presence of ddC on a fork-by-fork basis. We plotted the data as a percentage (Figure 6B–F) and cumulative percentage (Figure 6G) of forks at each ratio. Following ddC treatment, *BRCA1^−/−^* cells and *CtIP*^*S332A/−/−*^ were unable to maintain fork progression to the same extent as *wild-type* and *CtIP^+^*^*/−/−*^ cells. Likewise, *MRE11*^*D20A/−*^ mutant cells exhibited shorter CldU track lengths upon addition of ddC (Supplementary Figures S7C and S8). Our results suggest that BRCA1–CtIP and MRE11 are required for ensuring continued DNA synthesis following incorporation of the chain terminator ddCTP.

### BRCA1–CtIP and MRN complexes are required for restart of stalled replication forks

**Figure 7.**
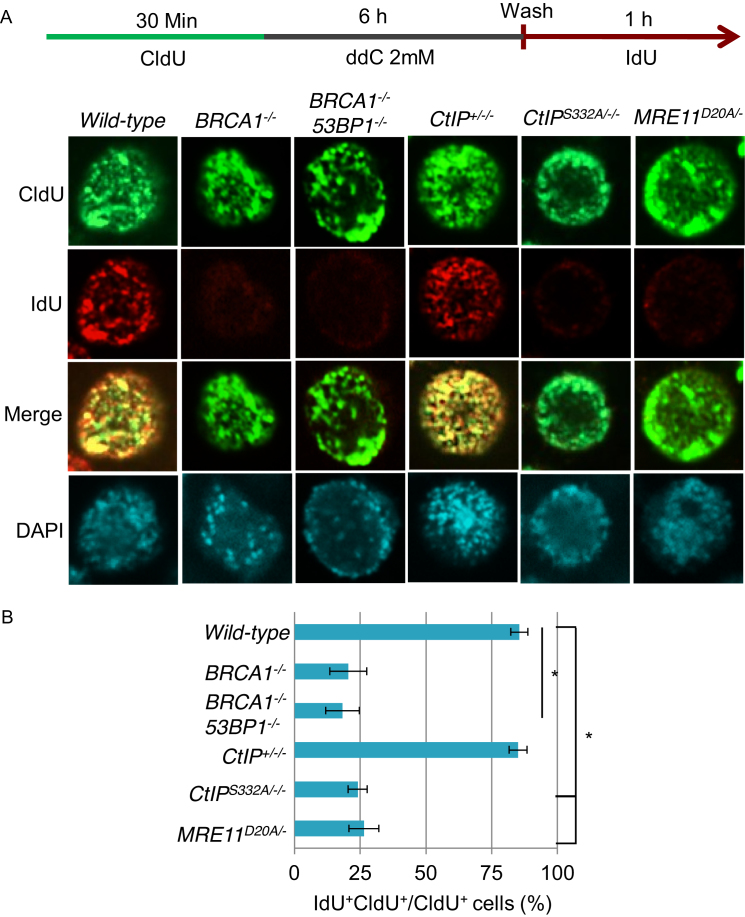
BRCA1, CtIP and MRE11 promote DNA restart of ddC-induced replication fork stalling. (**A**) Cells were pulsed with CldU, treated with 2 mM ddC for 6 h and released into IdU to analyze for DNA recovery. CldU and IdU were detected using specific primary and secondary antibodies in green and red, respectively. *BRCA1^−/−^, CtIP^S332A/−/−^* and nuclease-dead *MRE11^D20A/−^* mutants failed to resume DNA replication, whereas control and *CtIP^+/−/−^* cells demonstrated extensive DNA recovery as assessed by the overlapping CldU and IdU labeling. (**B**) Quantitation of the percentage of overlapping CldU and IdU labeling from A. At least 200 cells were counted per condition. Error bars represent standard deviations. CldU, 5-chloro-2-deoxyuridine; ddC, 2’,3’-di-deoxycytidine; IdU, 5-iodo-2-deoxyuridine.

Cellular sensitivity to ddC could be explained by delayed DNA-damage repair in the absence of BRCA1–CtIP complex and MRE11 nuclease activity, but it may also be linked to a defect in recovering from replicative stress. To determine whether BRCA1–CtIP and MRE11 play a role in DNA replication fork restart following ddC treatment, we employed a pulse labeling protocol to label ongoing replication with the CldU prior to ddC treatment, followed by pulsing with IdU to label restart of DNA replication after damage (39,40). We marked replicating cells by pulse labelling them with CldU, treated them with ddC for 6 h, and then allowed replication restart by removing the drug in the presence of IdU (Figure 7A). The results reveal significant recovery of DNA replication in both *wild-type* and *CtIP*^*+/−/−*^ cells in response to ddC as evidenced by extensive overlap of CldU and IdU labeling. However, *BRCA1^−/−^, CtIP*^*S332A*^*^/^*^*−/−*^ and *MRE11*^*D20A/−*^ mutant cells displayed severe replication restart defects under these conditions (Figure 7B). Remarkably, *53BP1^−/−^/BRCA1^−/−^* DT40 cells displayed similar defects in restarting replication forks following ddC treatment (Figure 7), further confirming an HR independent role for BRCA1 in response to ddC.

BRCA1, CtIP and MRE11 have been shown to be involved in processing and restarting stalled replication forks caused by hydroxyurea (HU) (38,40–42), which depletes deoxynucleotide pools and inhibits DNA replication (43,44). We next tested whether BRCA1–CtIP complex and the MRE11 nuclease activity were required for restart of stalled replication forks when exposed to HU. We marked replicating cells by pulse labelling them with CldU, treated them with HU for 6h, and then allowed replication to restart by removing the drug in the presence of IdU (Supplementary Figure S9A). The results revealed that *BRCA1^−/−^* and *MRE11*^*D20A/−*^ mutant cells displayed severe replication restart defects in response to HU, while *wild-type* and *CtIP*^*+/−/−*^ cells were able to restart replication under these conditions (Supplementary Figure S9B). Interestingly, *CtIP*^*S332A*^*^/^*^*−/−*^ cells displayed significant recovery of DNA replication in response to HU (Supplementary Figure S9B), indicating that BRCA1 and CtIP interaction is dispensable for the restart of HU-induced stalled replication forks.

In summary, phosphorylation-deficient *CtIP, BRCA1^−/−^* and nuclease dead *MRE11* mutant cells fail to resume replication after ddC treatment. These results suggest that the BRCA1–CtIP complex and MRE11 nuclease are required for ensuring continued DNA synthesis following incorporation of the chain terminator ddCTP.

### BRCA1, CtIP and MRE11 are required for cellular tolerance to nucleoside analogs in human cell lines

**Figure 8.**
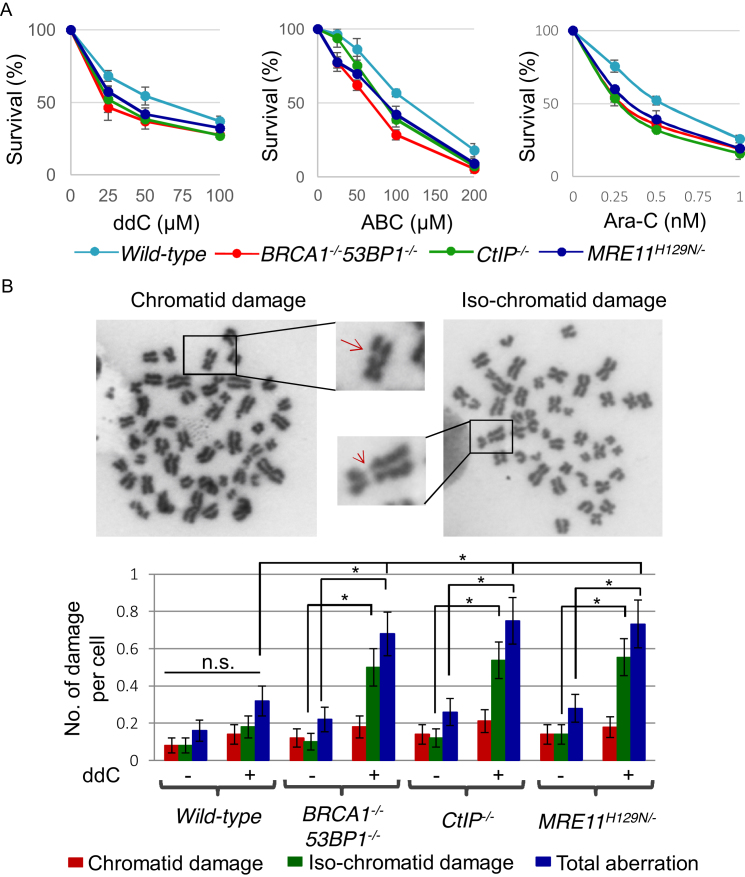
An important role of BRCA1, CtIP and MRE11 for cellular tolerance to nucleoside analogs in human TK6 cells. (**A**) Cell survival rate to the indicated agents was analyzed as described in Figure 1A. (**B**) Representative images of chromatid damage (arrow) and isochromatid damage (arrowhead) after ddC treatment (upper panel). Number of damages per mitotic cells in the indicated genotypes (lower panel) was calculated as described in Figure 4C.

To assess HR-independent functioning of BRCA1 in human cells, we measured the cellular sensitivity of *53BP1^−/−^/BRCA1^−/−^* TK6 mutant cells (45) to nucleoside analogs, as the deletion of the DNA damage response factor 53BP1 rescues HR deficiency of *BRCA1^−^*^*/−*^ cells (14,46). *BRCA1^−/−^53BP1^−/−^* human TK6 mutant cells exhibited considerably higher sensitivity to ddC, ABC and Ara-C in comparison with *wild-type* cells, consistent with the results in chicken DT40 cells (Figure 8A). Similarly, to investigate the role of the CtIP and MRE11 nuclease activity in human cells, we used conditionally disrupted *CtIP* and *MRE11* TK6 mutant cells (47), since the complete loss of CtIP or MRE11 is lethal to cells. Sensitivity data showed that *CtIP^−/−^* and *MRE11^H129N/−^* nuclease dead TK6 mutants also showed considerably higher sensitivity to ddC, ABC and Ara-C compared to *wild-type* cells (Figure 8A), indicating their involvement in cellular tolerance to CTNAs.

To further confirm sensitivity of TK6 cells to CTNAs, we measured the number of chromosomal aberrations in mitotic chromosome spreads at 24 hours post-ddC treatment (Figure 8B). Data show that ddC induced significantly increased numbers of isochromatid type chromosomal breaks in *BRCA1^−/−^53BP1^−/−^, CtIP^−/−^* and *MRE11^H129N/−^* nuclease dead TK6 mutants in comparison with *wild-type* cells (Figure 8B). These findings indicate that ddC affects chromosome integrity by inducing DNA damage through interfering with DNA replication during its incorporation by replicative DNA polymerases, leading to cell death. These observations suggest that BRCA1, CtIP and MRE11 contribute to cellular tolerance to CTNAs in human cells.

## DISCUSSION

In this study we have uncovered a role for BRCA1–CtIP complex and nuclease activity of MRE11 in maintaining replication fork progression upon ddC treatment. Replicative DNA polymerases frequently incorporate exogenously added CTNAs into the replicative DNA (7–9), which causes stalling or premature termination of DNA replication forks. Nucleoside analogs like ddC are used as antiviral agents because of their inability to be extended by viral DNA polymerases once incorporated into the viral DNA (7,48,49). Although human replicative DNA polymerases tend to limit the incorporation of ddC into nuclear DNA, exclusion of ddC from the large size of human genome is not absolute (9). Eventually, ddC can be incorporated into the genomic DNA and causes premature termination of replication forks. The increased sensitivity of nuclease dead *MRE11, BRCA1* and phosphorylation deficient *CtIP* mutants to ddC (Figures 1–3 and Supplementary Figure S2) and accumulation of large number of DSBs in these mutants (Figures 4 and 5, Supplementary Figures S5 and S6) that we observe, indicate that BRCA1–CtIP complex and MRE11 may be involved removing incorporated ddC from the replicative DNA. Such activities would be consistent with the removal of camptothecin-induced Top1 complex from 3’ ends of broken DNA (2,50).

Covalent bonds between topoisomerase 1 (Topo1) and the 3’ end of the single strand break (SSB) and between topoisomerase 2 (Topo2) and the 5’ end of the DSB are frequently formed (51). The anti-cancer agent, camptothecin (CPT) stabilizes Topo1-cleavage complexes. The covalently bound polypeptides must be eliminated from the DNA ends before being repaired. It has been reported that Topo2 cleavage complex can be removed from the DNA by the collaborative action of the MRN and BRCA1–CtIP complex (2,50,52). Thus, in addition to resection step in the HR pathway, MRN and BRCA1–CtIP complexes are involved in the elimination of covalent modification at the DNA ends. In the current study, we have shown that BRCA1–CtIP complex and MRE11 are also involved in the removal of chain terminating nucleoside analogs. Our findings support the activity of the BRCA1-CTIP complex, as *BRCA1^−/−^* and phosphorylation deficient *CtIP*^*S332A*^*^/^*^*−/−*^ cells, which is defective in interacting with BRCA1, are unable to maintain DNA replicative fork progression upon ddC exposure, relative to *wild-type* cells (Figure 6, Supplementary Figures S7 and S8). Moreover, our results support the involvement of the nuclease activity of MRE11, as the nuclease dead *MRE11^D20A/−^* cells are also unable to maintain DNA replicative fork progression in the presence of ddC (Figure 6, Supplementary Figures S7 and S8). In addition to the defective replication fork progression, *BRCA1^−/−^, CtIP*^*S332A*^*^/^*^*−/−*^ and *MRE11^D20A/−^* mutant cells displayed severe replication restart defects in response to CTNAs (Figure 7). The fact that *BRCA1^−/−^, CtIP*^*S332A*^*^/^*^*−/−*^ and *MRE11*^*D20A/−*^ cells fail to resume replication after ddC treatment could indicate that stalled replication forks are collapsed and are thus unable to restart replication in the absence of BRCA1–CtIP and MRN complexes. On the other hand, BRCA1–CtIP interaction is dispensable for the restart of HU-induced stalled replication forks (Supplementary Figure S9), which is consistent to a recent finding showing that the BRCA1 binding deficient CtIP-S327A mutant proteins rescued HU-induced fork degradation to a similar extent as CtIP-WT protein (42). It has been shown that BRCA1 and CtIP interact with MRN complex and this interaction is largely dependent on the interaction of CtIP with the BRCT domains at the C terminus of BRCA1 (53–56). BRCA1, CtIP, and MRN protein complex forms when cells enter the S and G_2_ phases of the cell cycle (54). Recent evidence shows that BRCA1 contributes to the removal of pathological TOP2ccs possibly through the nuclease activity of MRE11 (45). We therefore conclude that the BRCA1–CtIP complex promotes MRE11 nuclease activity to remove incorporated ddC from the nascent DNA to maintain genome stability (Supplementary Figure S10). We propose that BRCA1-CTIP and MRE11 prepare DNA ends for further repair, much as they do in the repair of Top1-bound complexes at DSBs (2).

MRE11 also plays multiple roles in genome maintenance, including DSB resection, restart of stalled DNA replication forks, and activation of DNA damage checkpoint (57). Experiments with hydroxyurea, which causes depletion of free deoxynucleotide pool and inhibits DNA replication, have successfully defined S-phase checkpoint in the yeast genetic study (58). However, physiological relevance of the same experiment with vertebrate cells is unclear. To investigate the role of MRE11, previous studies exposed cells to hydroxyurea for 2 h or more, and measured the restart of replication forks in MRE11-depleted *S. cerevisiae* and mammalian cells (58–61). In the current study, we have measured the effect of ddC immediately after addition of ddC into the cells and investigated the MRE11 nuclease activity in the removal of incorporated ddC from the replicative DNA. In addition to hydroxyurea, alkylating agents and UV have been used for increasing replication stress and investigating the role of MRE11 in counteracting the stress (40,62). These studies revealed the role of MRE11 in HR for preventing replication fork collapse under the replication stress conditions. Experiments with alkylating agents and UV address the capability of TLS but not necessarily HR (21,22), which address a question very different from that which we address here. Like TDP1, the Rad32^MRE11^ nuclease activity of *Schizosaccharomyces pombe* is also involved in the elimination of Top1 from 3′ DNA ends (38). In the current study, we have shown that MRE11 nuclease activity is involved in the removal of incorporated ddC from the replicative DNA. As with the two paths to eliminate polypeptides covalently bound at the end of DSBs (2), eukaryotic cells possess two redundant mechanisms to remove incorporated CTNAs from the 3′ end of nascent DNA. Firstly, incorporated CTNAs can be eliminated by a tyrosyl-DNA phosphodiesterase such as TDP1 (8). On the other hand, direct removal of incorporated CTNAs can be achieved by the nucleolytic activities of MRN–CtIP/BRCA1 complexes (Supplementary Figure S10). In summary, we here show persuasive evidence that MRE11 and the collaborative action of BRCA1 and CtIP play critical role in the nuclease-dependent removal of incorporated ddC from the replicating DNA. It is tempting to speculate that the existence of an endogenous activity to remove exogenously added chain terminating nucleosides supports the occurrence of natural chain terminating nucleosides or poorly extended precursors, possibly arising by damage to NTP/dNTP pools (48–54).

## Supplementary Material

Supplementary Data
