# Supplementary material for: CtIP-BRCA1 complex and MRE11 maintain replication forks in the presence of chain terminating nucleoside analogs
Source: Nucleic Acids Res. 2019 Jan 18;47(6):2966–80. doi: 10.1093/nar/gkz009 (PMC6451104; doi:10.1093/nar/gkz009)
Supplement: Supplementary Data [file gkz009_supplemental_files.pdf]

Supplementary Information

CtIP-BRCA1 complex and MRE11 maintain replication forks in the presence of chain terminating nucleoside analogs

Mohiuddin Mohiuddin[1], Md Maminur Rahman[2], Julian E. Sale[3], and Christopher E. Pearson[1,4*]

[1]Program of Genetics and Genome Biology, The Hospital for Sick Children, Toronto, Ontario M5G 0A4, Canada.
[2]Department of Radiation Genetics, Kyoto University Graduate School of Medicine, Yoshida Konoe, Sakyo-ku, Kyoto 606-8501, Japan.
[3]Medical Research Council Laboratory of Molecular Biology, Hills Road, Cambridge, CB2 0QH, UK.
[4]The Department of Molecular Genetics, University of Toronto, Toronto, ON M5S 1A8, Canada.

*To whom correspondence should be addressed: Christopher E. Pearson (E-mail: cepearson.sickkids@gmail.com).

Supplementary Table 1. List of cell lines used in this study

Genotype	Parental Cell line	References
CtIP$^{+/-/-}$	Chicken DT40	(1)
CtIP$^{S332A/-/-}$	Chicken DT40	(1)
BRCA1$^{-/-}$	Chicken DT40	(2)
BRCA1$^{-/-}$*CtIP*$^{S332A/-/-}$	Chicken DT40	(1)
53BP1$^{-/-}$	Chicken DT40	(3)
BRCA1$^{-/-}$*53BP1*$^{-/-}$	Chicken DT40	(4)
BRCA2$^{-/-}$	Chicken DT40	(5)
MRE11$^{-/D20A}$	Chicken DT40	Fig. S3
PARP1$^{-/-}$	Chicken DT40	(6)
TDP1$^{-/-}$*TDP2*$^{-/-}$	Chicken DT40	(7)
MRE11$^{H63S/-}$	Chicken DT40	Fig. S3
BRCA1$^{-/-}$*MRE11*$^{H63S/-}$	Chicken DT40	Fig. S3
NBS1$^{-/-/-}$	Chicken DT40	(8)
BRCA1$^{-/-}$*53BP1*$^{-/-}$	Human TK6	(9)
CtIP$^{-/-}$	Human TK6	(10)
MRE11$^{-/H129N}$	Human TK6	(10)

Supplementary Figure legends

Supplementary Figure S1: Disruption of 53BP1 reverses the HR phenotype of BRCA1$^{-/-}$ chicken DT40 cells. (A) Proliferation rate of the indicated genotypes. Y-axis and X-axis represent the relative number of cells and the time respectively. (B) Representative images for the accumulation of RAD51 to the DNA damage sites following 2Gy gamma irradiation. (C) Histogram shows the number of RAD51 foci per cells following gamma irradiation. Accumulation of RAD51 at DNA damage sites is reversed in the *53BP1*$^{-/-}$*BRCA1*$^{-/-}$ mutant cells. The *P*-value for Student's t-test was *$p<0.05$.

Supplementary Figure S2: Sensitivity profiles of the indicated chain terminating

nucleoside analogs in the selected DNA repair deficient DT40 cells. The colony survival was measured as in Fig. 1. The relative sensitivity of each isogenic mutant chicken DT40 cell line was compared to that of the wild-type DT40 cell line. Negative (left) or positive (right) scores indicate that the cell line was either sensitive or resistant to the specified nucleoside-analog. Each bar length reflects the degree of sensitivity or resistance to the drug (log2 units). Error bars show the SD of the mean for three independent assays.

Supplementary Figure S3: The validation of nuclease dead *MRE11* mutants. (A) Site directed mutagenesis of $MRE11^{D20A/-}$ mutant was confirmed by direct sequencing from genomic DNA. Reverse primer was used to sequence antisense strand of *MRE11*. Sequence for the complementary strands were provided. Sequence chromatograph covering the MRE11 codon, which was changed from Asp (D) in the wild-type clone to Ala (A) in the $MRE11^{D20A/-}$ clone. (B) Site directed mutagenesis of $MRE11^{H63S/-}$ and $BRCA1^{-/-}MRE11^{H63S/-}$ mutants were confirmed as indicated in figure S3A. Sequence chromatograph covering the MRE11 codon, which were changed from His (H) in the wild-type clone to Ser (S) in the $MRE11^{H63S/-}$ and $BRCA1^{-/-}MRE11^{H63S/-}$ clones.

Supplementary Figure S4: An important role of NBS1 for cellular tolerance to nucleoside analogs in DT40 cells. Liquid-culture cell survival in the presence of the indicated genotoxic agents. Survival rate to the indicated agents was analyzed as described in Figure 1A.

Supplementary Figure S5: $BRCA1^{-/-}$, $CtIP^{S332A/-/-}$ and nuclease-dead $MRE11^{D20A/-}$ cells exhibited significantly increased number of γH2AX foci following ddC treatment. (A) Representative fluorescence microscopic images of γH2AX foci in the indicated cell lines before and 9 h after exposure of 100 μM ddC. (B) The bar graph (upper panel) represents mean and SD of % γH2AX-foci positive cells (> seven foci per cell) before and 9 h after exposure of 100 μM ddC in three independent experiments. At least 100 cells were counted per condition in each experiment. The box and whisker plot (lower panel) represents median number of γH2AX-foci per cells. Whiskers the first and

third quartiles. Statistical analyses were performed by student's t-test (*p<0.01).

Supplementary Figure S6: *BRCA1$^{-/-}$*, *BRCA1$^{-/-}$53BP1$^{-/-}$*, *CtIP$^{S332A/-/-}$* and nuclease-dead *MRE11$^{D20A/-}$* mutants exhibit increased number of ddC-induced DNA double strand breaks. Representative comet images of *wild-type*, *BRCA1$^{-/-}$*, *BRCA1$^{-/-}$53BP1$^{-/-}$*, *CtIP$^{+/-/-}$*, *CtIP$^{S332A/-/-}$* and nuclease-dead *MRE11$^{D20A/-}$* DT40 cells with or without ddC treatment for 2 h.

Supplementary Figure S7: CtIP, BRCA1 and MRE11 are dispensable to maintain the velocity of unperturbed DNA synthesis. (A) Schematic of treatment with ddC and pulse labeling with IdU and CldU are shown (Upper panel). Representative single forks for the indicated genotypes (Lower panel). (B) Replication fork lengths were obtained by converting the IdU track sizes in µM to kb and analyzed in the indicated cell types. IdU track lengths are calculated by dividing the track lengths by the labeling time and depicted above the track lengths. More than 100 forks were calculated in each cell types. Statistical analysis was done by Student's t-test (n.s.: not significant). (C) Replication fork lengths were obtained by converting the CldU track sizes in µM to kb and analyzed in the indicated cell types. CldU track lengths are calculated by dividing the track lengths by the labeling time and depicted above the track lengths. More than 100 forks were calculated in each cell types. The *P*-values for Student's t-test were *p<0.001 and **p<0.05.

Supplementary Figure S8: Representative images for the defective progression of replication forks on ddc treatment. (A-E) Representative images for DNA fibres in the ddC treated WT (A), *CtIP$^{+/-/-}$* (B), *CtIP$^{S332A/-/-}$* (C), *BRCA1$^{-/-}$* (D) and *MRE11$^{-/D20A}$* (E) chicken DT40 cell lines.

Supplementary Figure S9: BRCA1 and MRE11 promote DNA restart of HU-induced replication fork stalling. (A) Cells were pulsed with CldU, treated with 2 mM HU for 6 h and released into IdU to analyze for DNA recovery. Replication restart rate was analyzed as described in Figure 7.

Supplementary Figure S10: Model for the restart of ddC-induced stalled replication fork. Chain terminating nucleoside analog such as 2'3' dideoxycytidine (ddC), causes stalling or premature termination of DNA replication forks upon incorporation into the DNA. There are two distinct pathways to re-stall the replication forks. In the first pathway, TDP1 –dependent repair excises the CTNAs blocking the 3'-ends directly and maintains the progression of replication forks (11). In the second pathway, MRN complex and the collaborative action of BRCA1 and CtIP play critical role in the nuclease-dependent removal of incorporated CTNAs from the replicative DNA.

References

1. Nakamura,K., Kogame,T., Oshiumi,H., Shinohara,A., Sumitomo,Y., Agama,K., Pommier,Y., Tsutsui,K.M., Tsutsui,K., Hartsuiker,E., *et al.* (2010) Collaborative action of Brca1 and CtIP in elimination of covalent modifications from double-strand breaks to facilitate subsequent break repair. *PLoS Genet.*, **6**, e1000828.
2. Martin,R.W., Orelli,B.J., Yamazoe,M., Minn,A.J., Takeda,S. and Bishop,D.K. (2007) RAD51 up-regulation bypasses BRCA1 function and is a common feature of BRCA1-deficient breast tumors. *Cancer Res.*, **67**, 9658–9665.
3. Nakamura,K., Sakai,W., Kawamoto,T., Bree,R.T., Lowndes,N.F., Takeda,S. and Taniguchi,Y. (2006) Genetic dissection of vertebrate 53BP1: A major role in non-homologous end joining of DNA double strand breaks. *DNA Repair (Amst).*, **5**, 741–749.
4. Hoa,N.N., Kobayashi,J., Omura,M., Hirakawa,M., Yang,S.H., Komatsu,K., Paull,T.T., Takeda,S. and Sasanuma,H. (2015) BRCA1 and CtIP are both required to recruit Dna2 at double-strand breaks in homologous recombination. *PLoS One*, **10**, e0124495.
5. Hatanaka,A., Yamazoe,M., Sale,J.E., Takata,M., Yamamoto,K., Kitao,H., Sonoda,E., Kikuchi,K., Yonetani,Y. and Takeda,S. (2005) Similar effects of Brca2 truncation and Rad51 paralog deficiency on immunoglobulin V gene diversification in DT40 cells support an early role for Rad51 paralogs in homologous recombination. *Mol. Cell. Biol.*, **25**, 1124–1134.

6. Fortune,J.M. and Osheroff,N. (2000) Topoisomerase II as a target for anticancer drugs: when enzymes stop being nice. *Prog. Nucleic Acid Res. Mol. Biol.*, **64**, 221–53.
7. Zeng,Z., Sharma,A., Ju,L., Murai,J., Umans,L., Vermeire,L., Pommier,Y., Takeda,S., Huylebroeck,D., Caldecott,K.W., *et al.* (2012) TDP2 promotes repair of topoisomerase I-mediated DNA damage in the absence of TDP1. *Nucleic Acids Res.*, **40**, 8371–8380.
8. Nakahara,M., Sonoda,E., Nojima,K., Sale,J.E., Takenaka,K., Kikuchi,K., Taniguchi,Y., Nakamura,K., Sumitomo,Y., Bree,R.T., *et al.* (2009) Genetic evidence for single-strand lesions initiating Nbs1-dependent homologous recombination in diversification of Ig V in chicken B lymphocytes. *PLoS Genet.*, **5**, e1000356.
9. Sasanuma H, Tsuda M, Morimoto S, Saha LK, Rahman MM, Kiyooka Y, Fujiike H, Cherniack AD, Itou J, Callen Moreu E, Toi M, Nakada S, Tanaka H, Tsutsui K, Yamada S, Nussenzweig A,T.S. (2018) BRCA1 ensures genome integrity by eliminating estrogen-induced pathological topoisomerase II-DNA complexes. *Proc. Natl. Acad. Sci.*, **115**, 10642-51.
10. Hoa,N.N., Akagawa,R., Yamasaki,T., Hirota,K., Sasa,K., Natsume,T., Kobayashi,J., Sakuma,T., Yamamoto,T., Komatsu,K., *et al.* (2015) Relative contribution of four nucleases, CtIP, Dna2, Exo1 and Mre11, to the initial step of DNA double-strand break repair by homologous recombination in both the chicken DT40 and human TK6 cell lines. *Genes to Cells*, **20**, 1059-76.
11. Huang,S.Y.N., Murai,J., Dalla Rosa,I., Dexheimer,T.S., Naumova,A., Gmeiner,W.H. and Pommier,Y. (2013) TDP1 repairs nuclear and mitochondrial DNA damage induced by chain-terminating anticancer and antiviral nucleoside analogs. *Nucleic Acids Res.*, **41**, 7793–7803.

Mohiuddin et al., Supplementary Figure S1

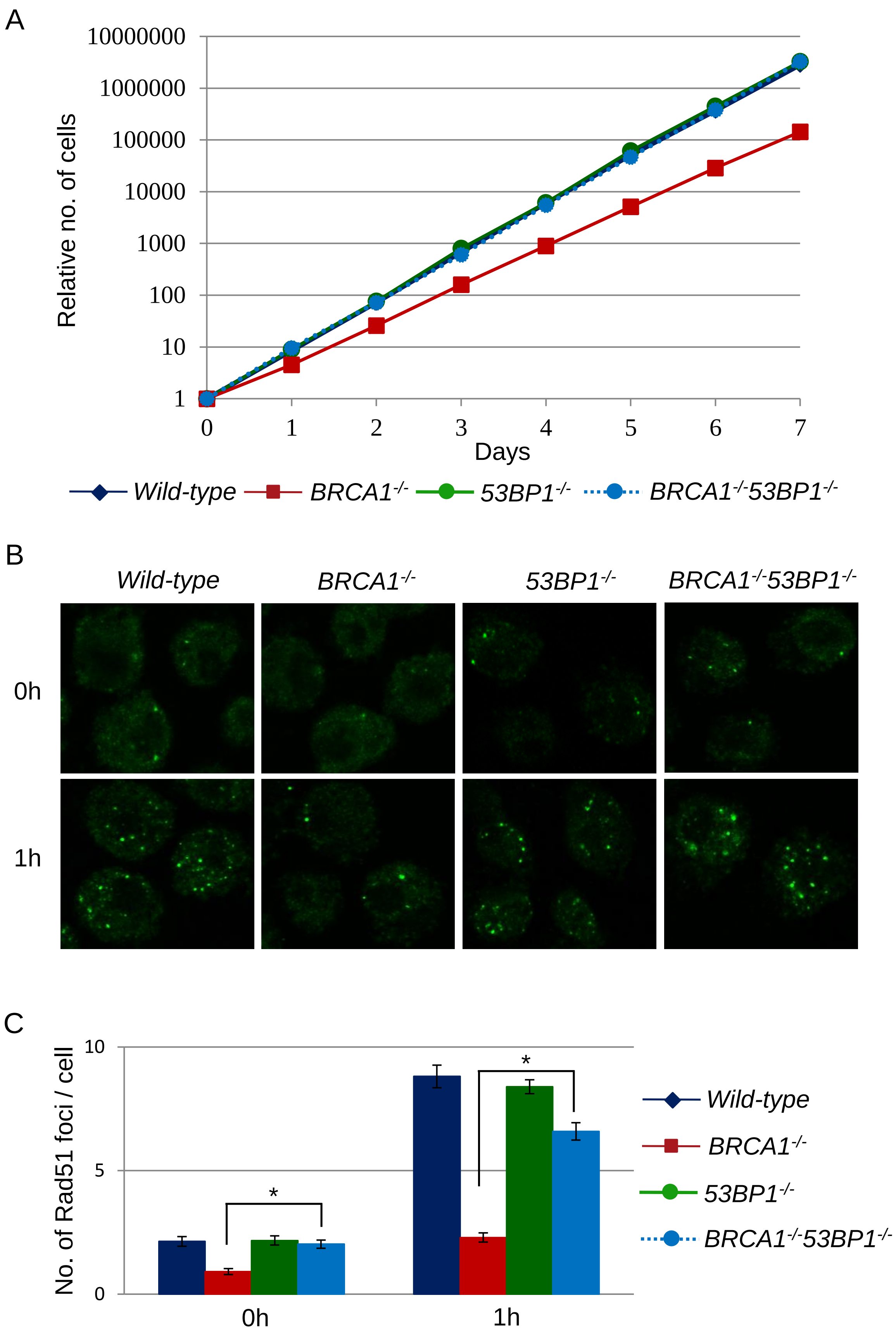

Mohiuddin et al., Supplementary Figure S2

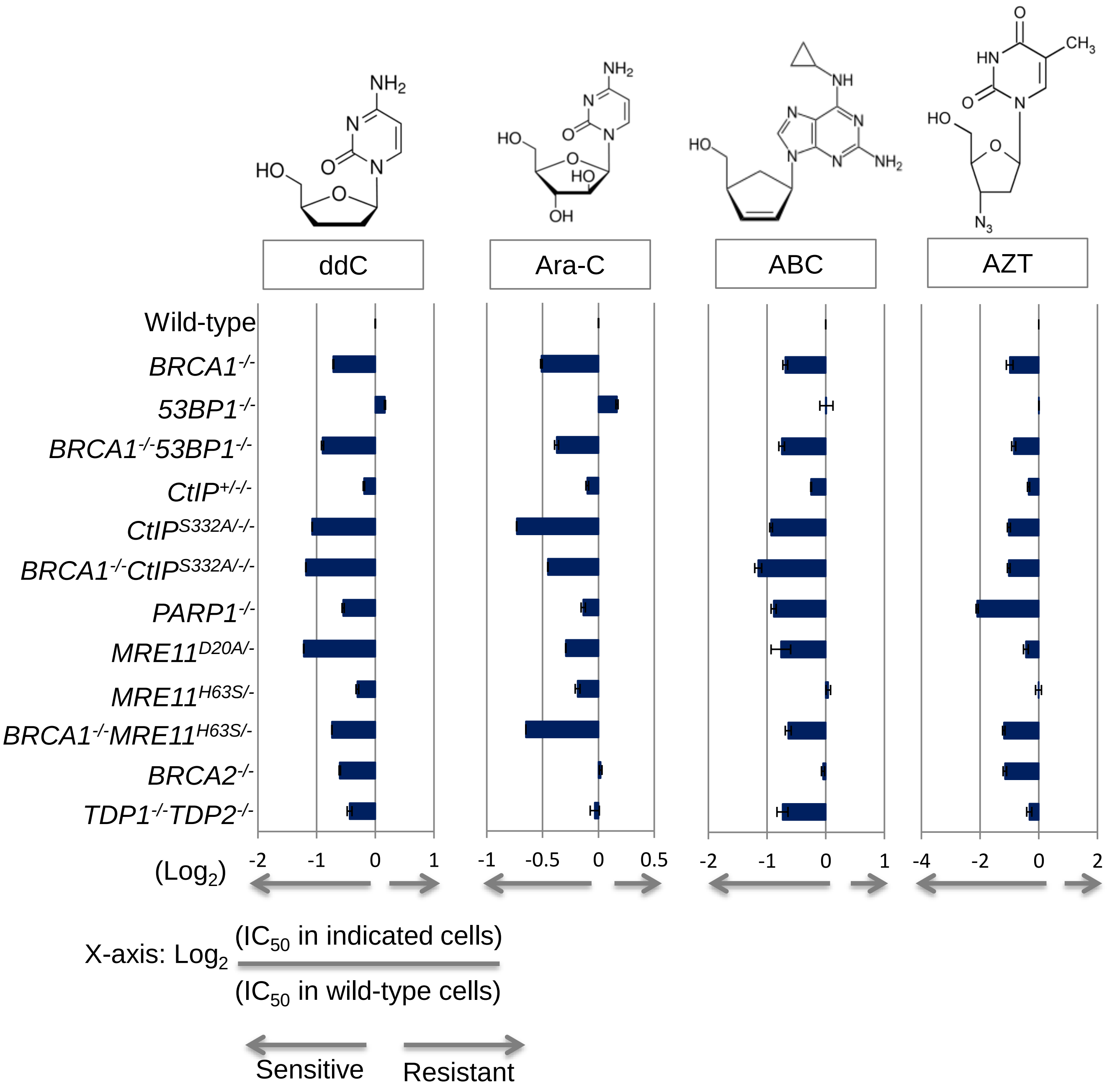

A

Wild-type
(Antisense strand)

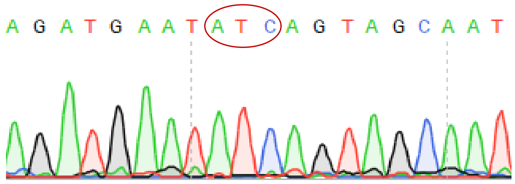

Complementary Strand

ATTGCTACTGATATTCATCT

MRE11$^{D20A/-}$
(Antisense strand)

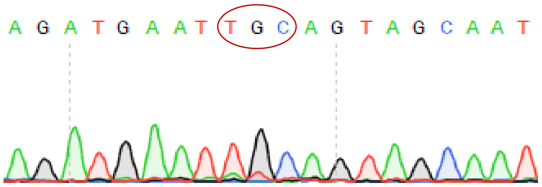

Complementary Strand

ATTGCTACTGCAATTCATCT

B

Wild-type
(Antisense strand)

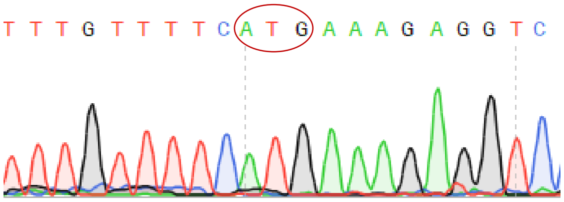

Complementary Strand

GACCTCTTTCATGAAAACAAA

MRE11$^{H63S/-}$
(Antisense strand)

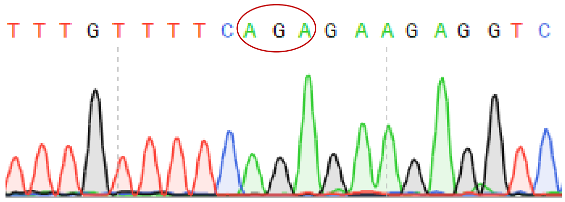

Complementary Strand

GACCTCTTCTCTGAAAACAAA

BRCA1$^{-/-}$*MRE11*$^{H63S/-}$
(Antisense strand)

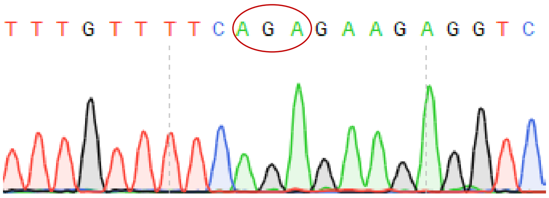

Complementary Strand

GACCTCTTCTCTGAAAACAAA

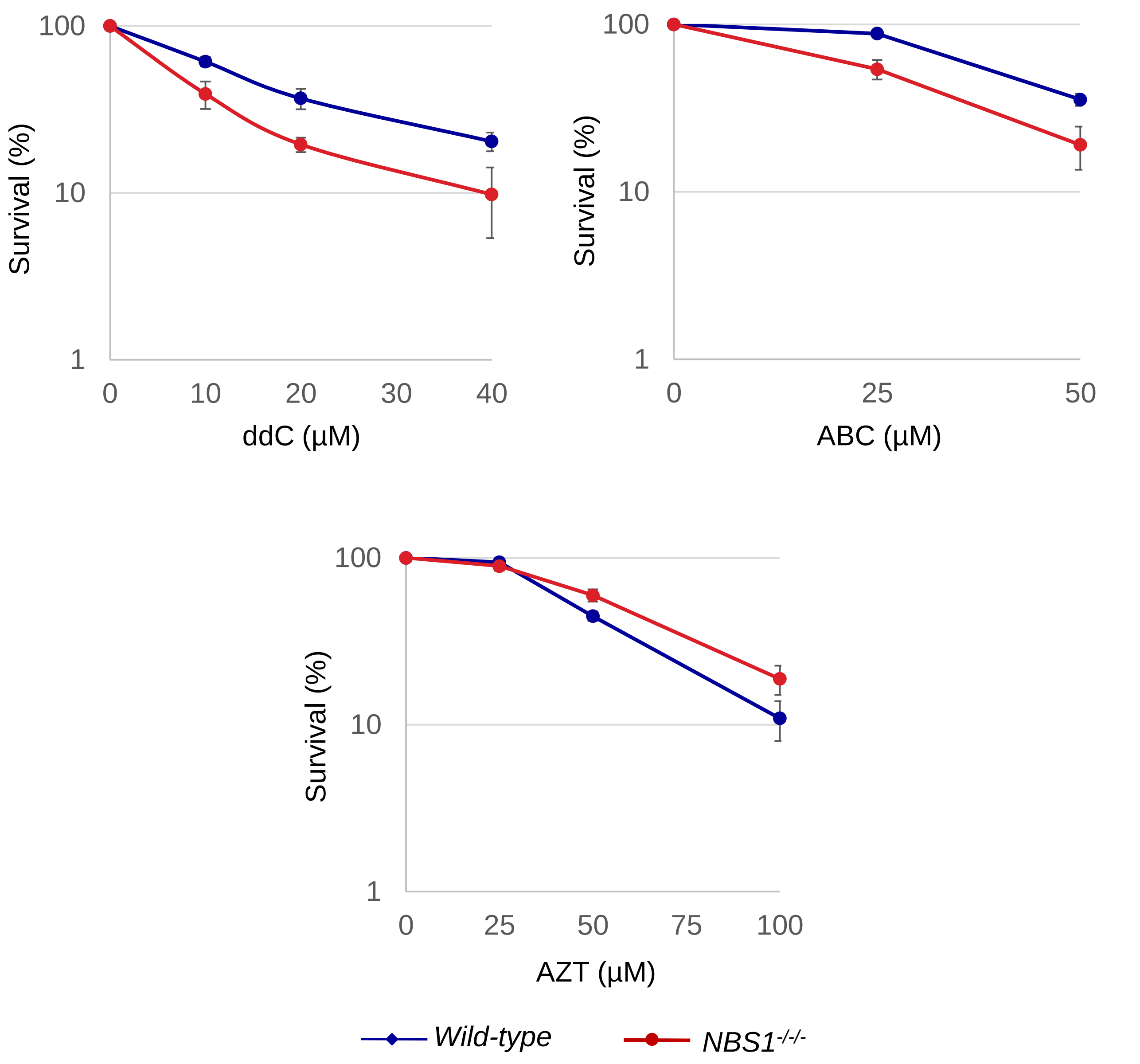

100
10
1
Survival (%)
0
10
20
30
40
ddC (µM)
100
10
1
Survival (%)
0
25
50
ABC (µM)
100
10
1
Survival (%)
0
25
50
75
100
AZT (µM)
Wild-type
NBS1-/-/-

A

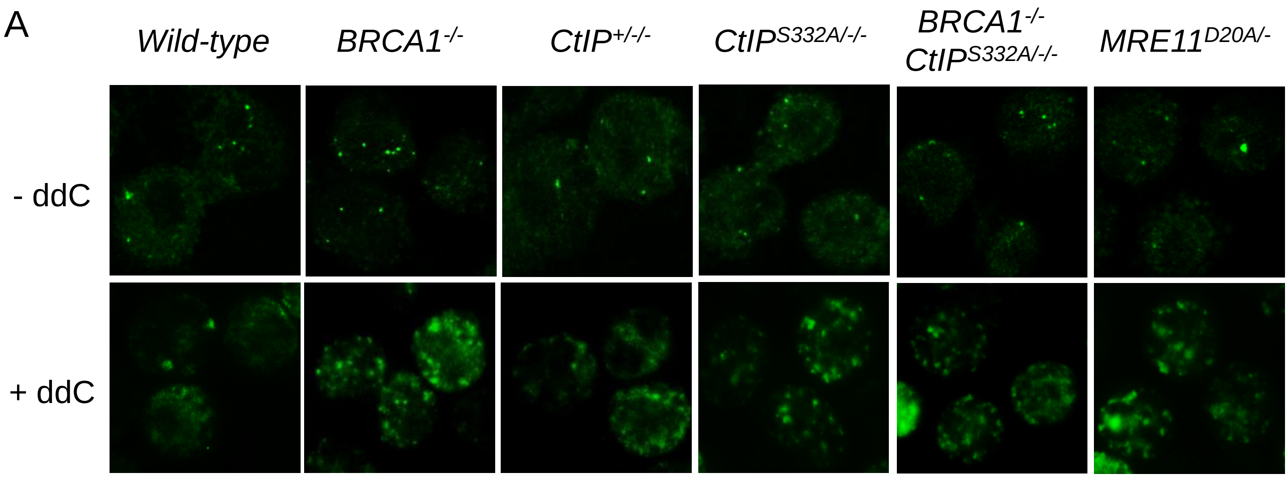
Wild-type
BRCA1-/-
CtIP+/-/-
CtIPS332A/-/-
BRCA1-/- CtIPS332A/-/-
MRE11D20A/-
- ddC
+ ddC

B

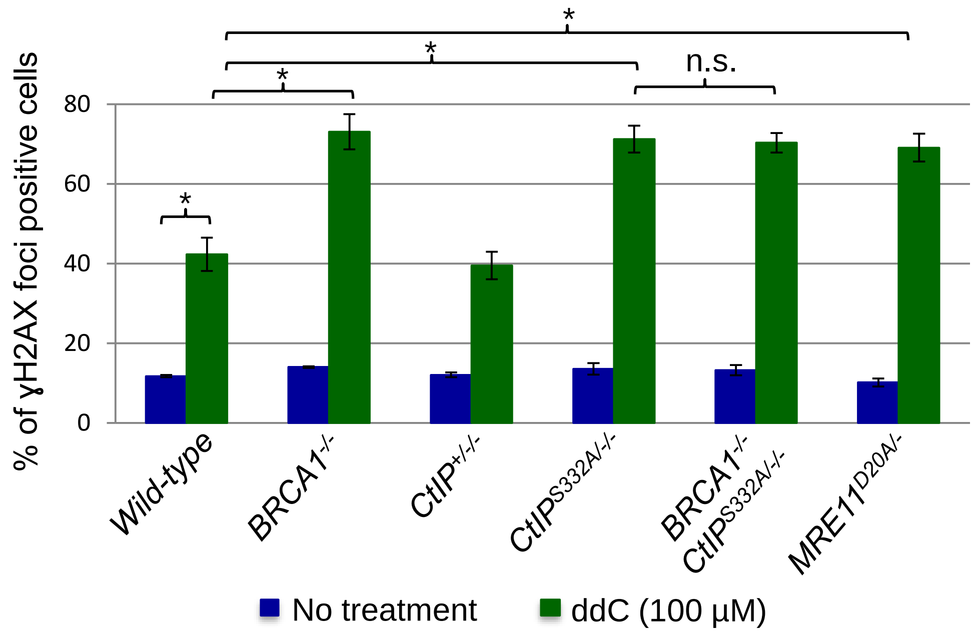
% of γH2AX foci positive cells
80
60
40
20
0
*
*
*
*
n.s.
Wild-type
BRCA1-/-
CtIP+/-/-
CtIPS332A/-/-
BRCA1-/- CtIPS332A/-/-
MRE11D20A/-
No treatment
ddC (100 µM)

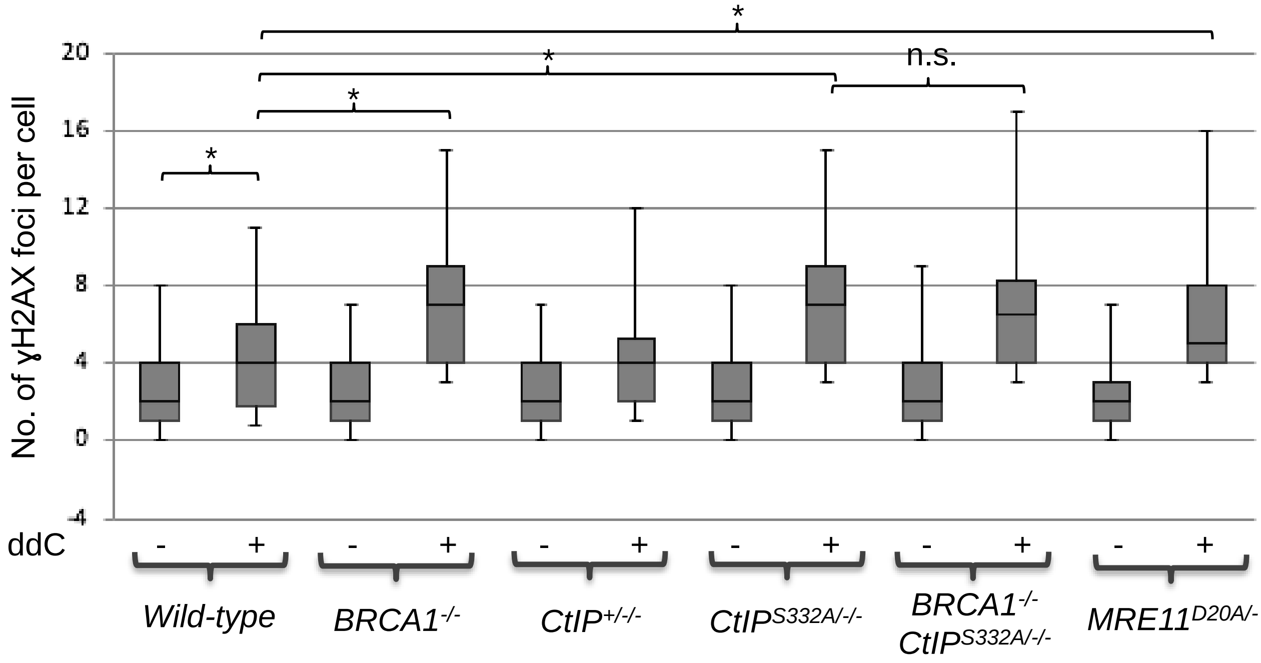
No. of γH2AX foci per cell
20
16
12
8
4
0
-4
*
*
*
*
n.s.
ddC
- +
- +
- +
- +
- +
- +
Wild-type
BRCA1-/-
CtIP+/-/-
CtIPS332A/-/-
BRCA1-/- CtIPS332A/-/-
MRE11D20A/-

Mohiuddin et al., Supplementary Figure S6

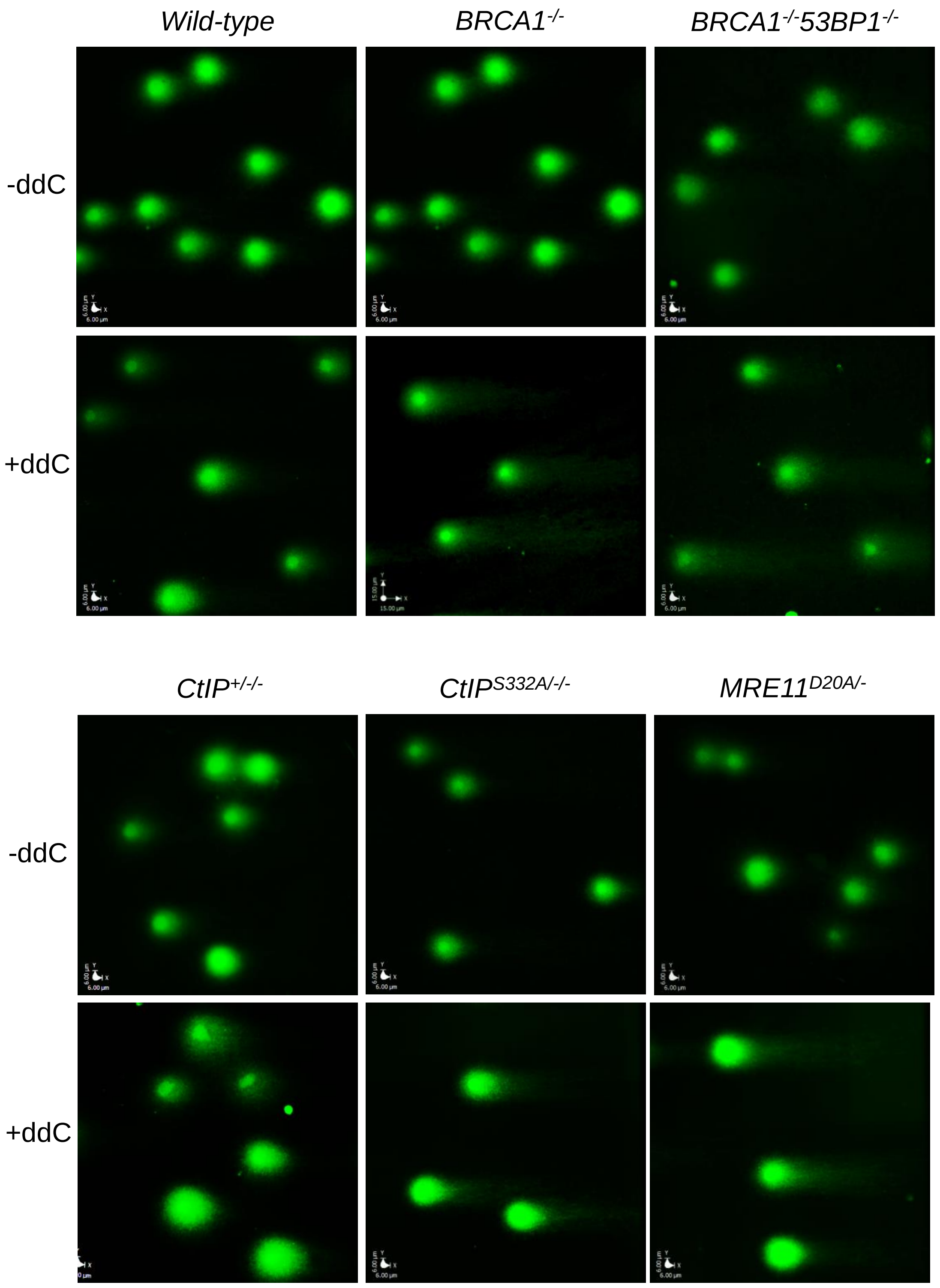

Mohiuddin et al., Supplementary Figure S7

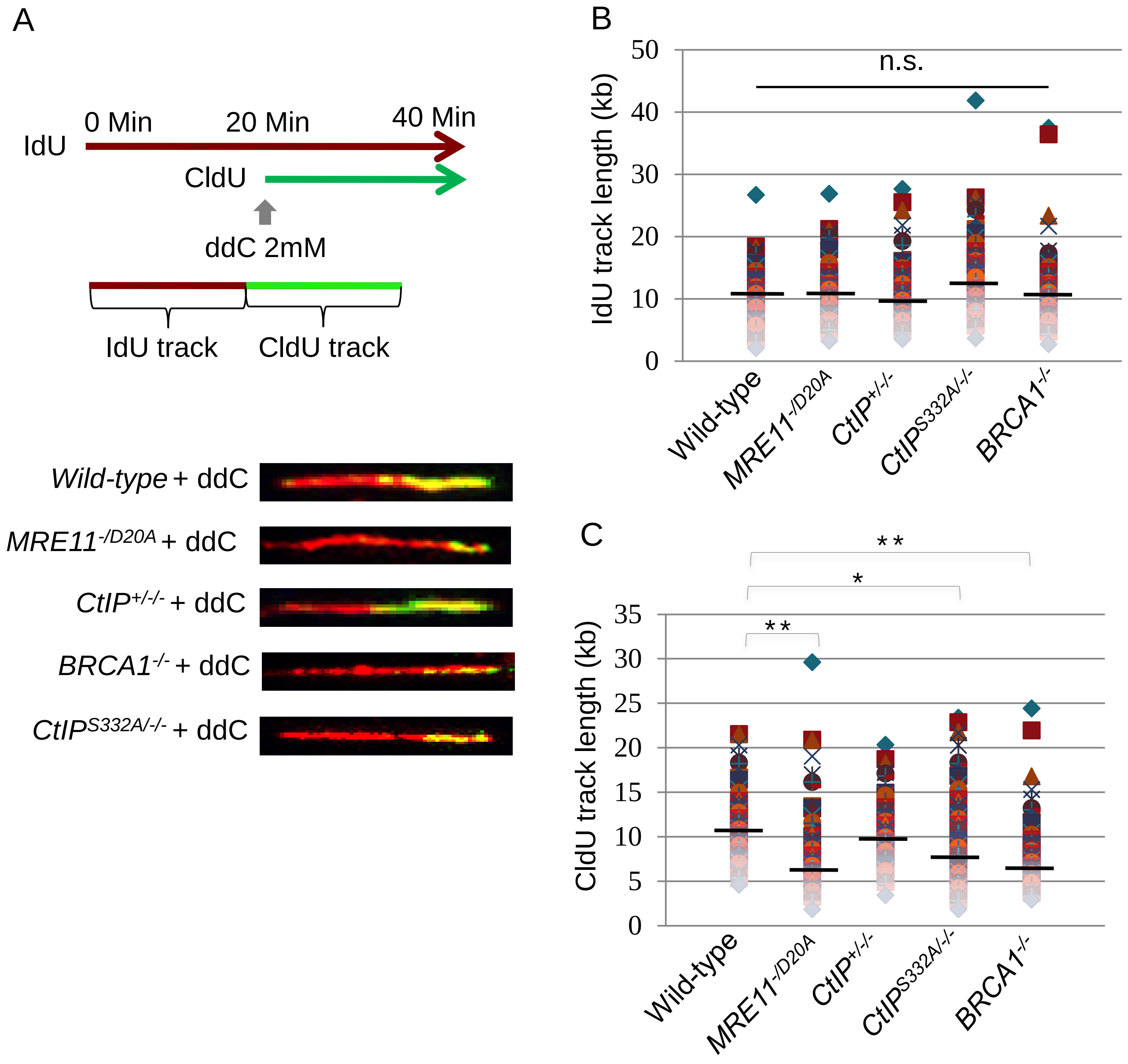

Mohiuddin et al., Supplementary Figure S8

A *Wild-type* + ddC

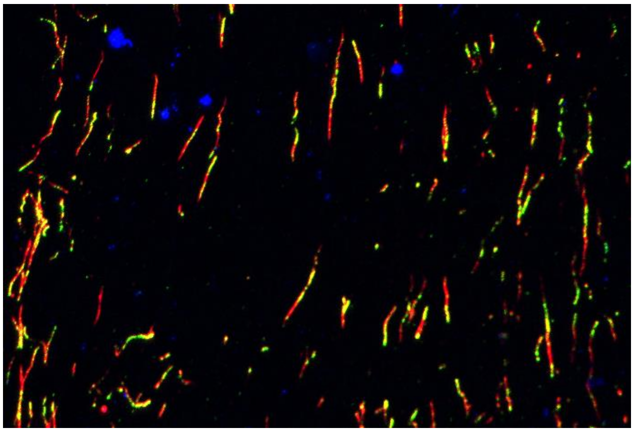

B $CtIP^{+/-/-}$ + ddC

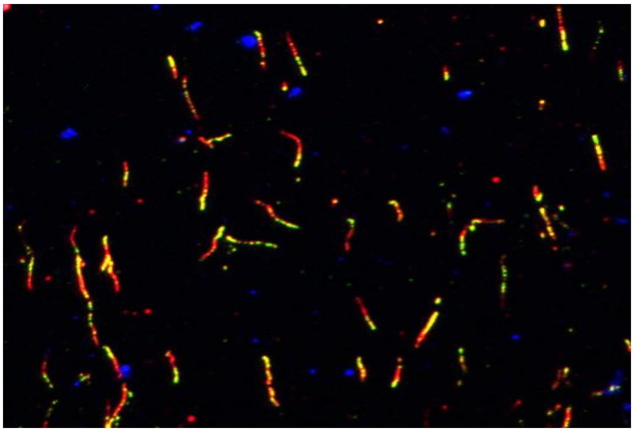

C $CtIP^{S332A/-/-}$ + ddC

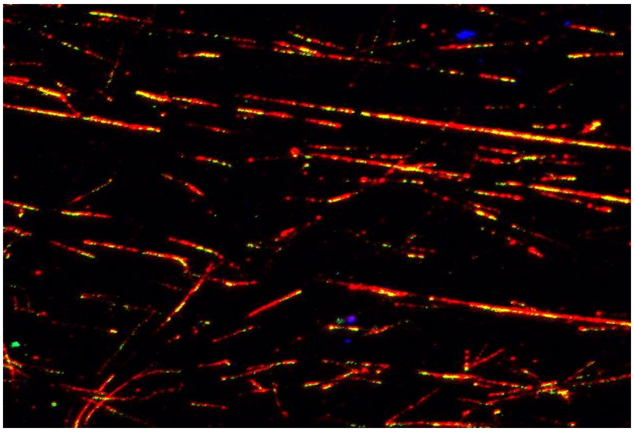

D $BRCA1^{-/-}$ + ddC

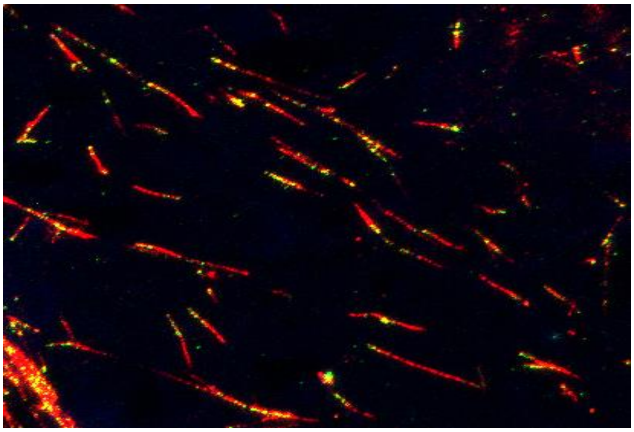

E $MRE11^{-/D20A}$ + ddC

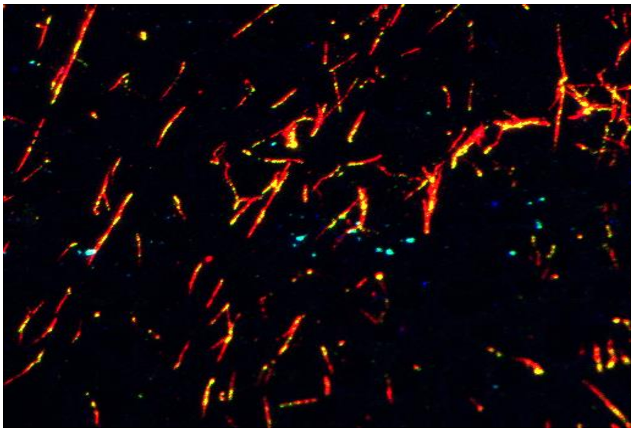

Mohiuddin et al., Supplementary Figure S9

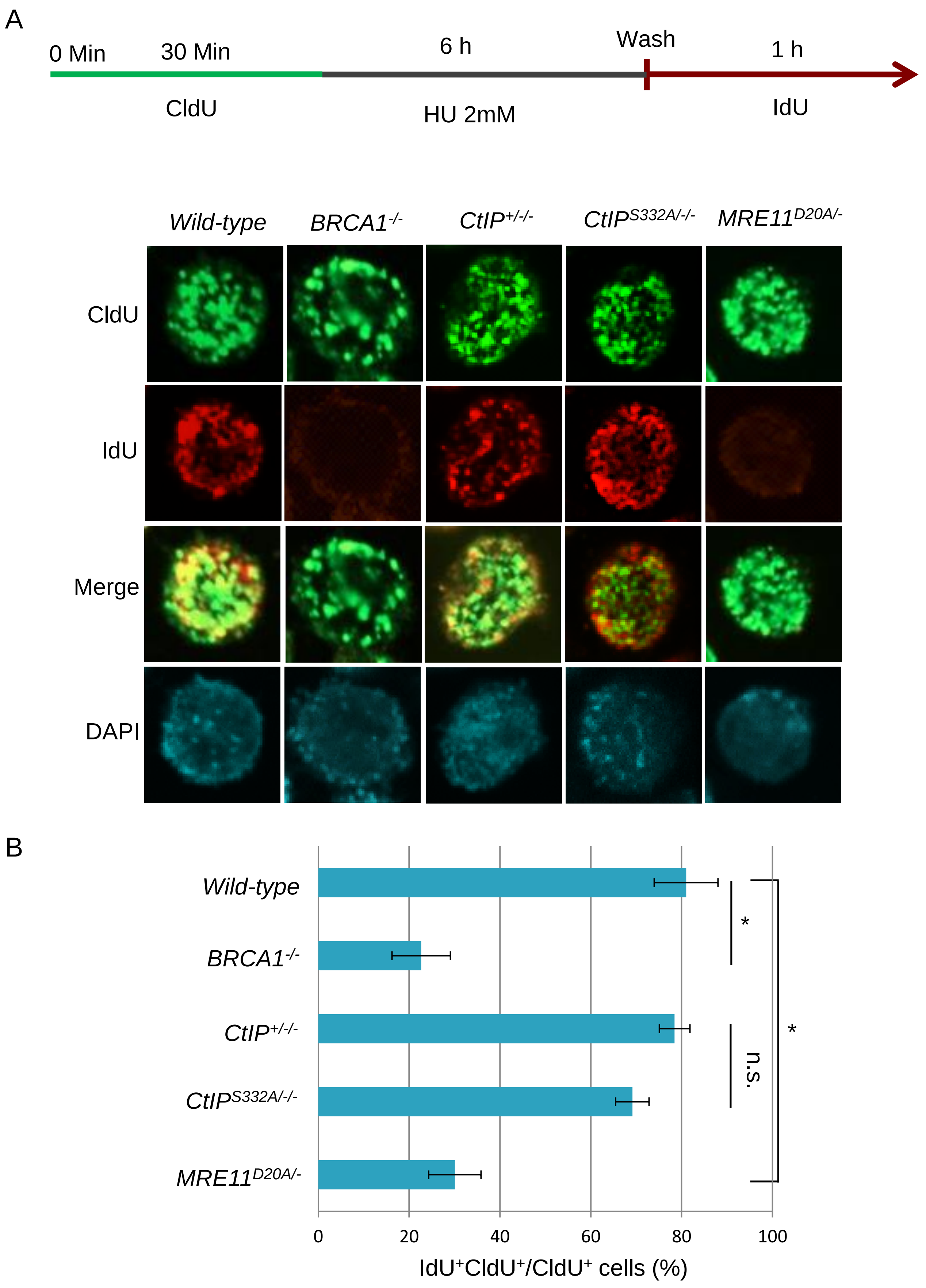

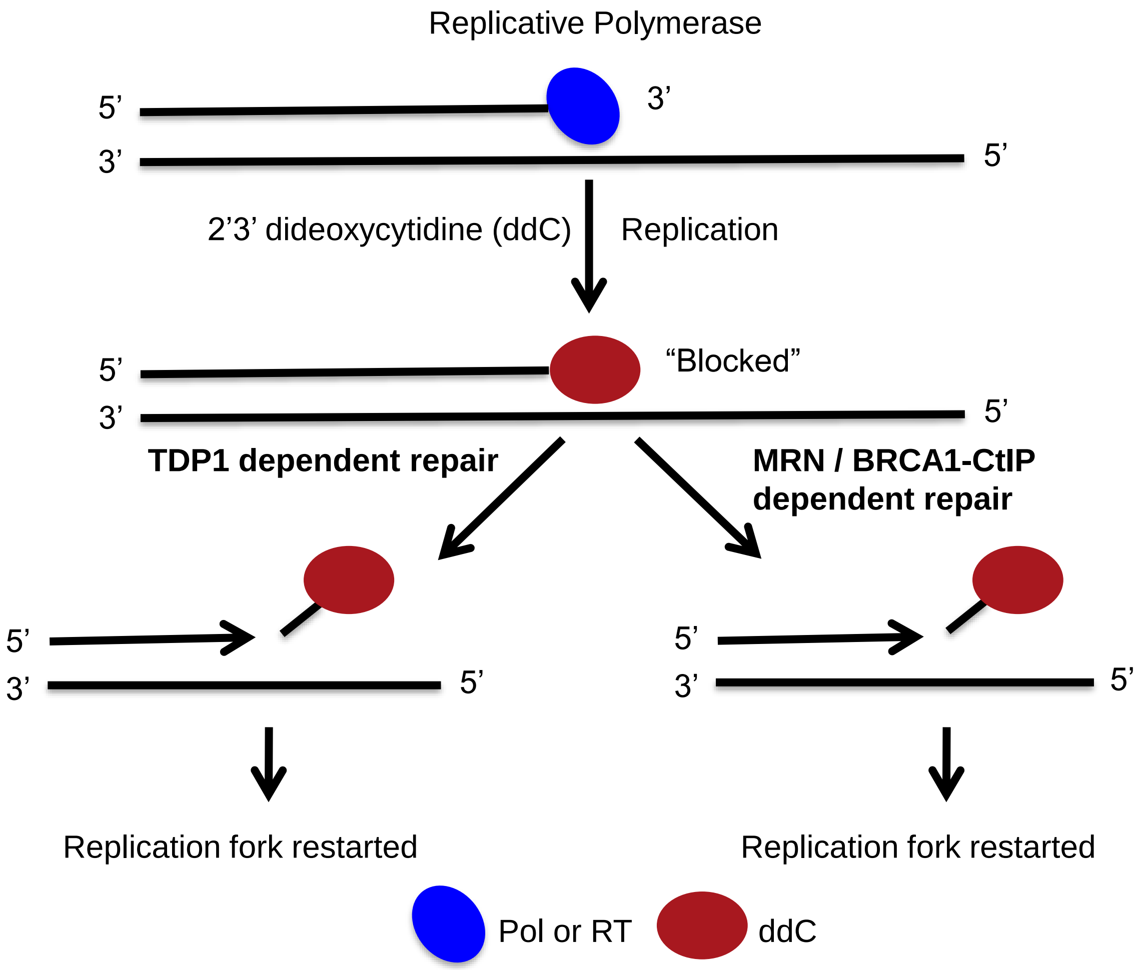
Replicative Polymerase
5'
3'
3'
5'
2'3' dideoxycytidine (ddC)
Replication
5'
3'
"Blocked"
5'
TDP1 dependent repair
MRN / BRCA1-CtIP dependent repair
5'
3'
5'
5'
3'
5'
Replication fork restarted
Replication fork restarted
Pol or RT
ddC
